# Faecal Bacteriome and Metabolome Profiles Associated with Decreased Mucosal Inflammatory Activity Upon Anti-TNF Therapy in Paediatric Crohn’s Disease

**DOI:** 10.1093/ecco-jcc/jjad126

**Published:** 2023-08-01

**Authors:** Jakub Hurych, Anna Mascellani Bergo, Tereza Lerchova, Lucie Hlinakova, Michal Kubat, Hana Malcova, Dita Cebecauerova, Jan Schwarz, Eva Karaskova, Tomas Hecht, Radim Vyhnanek, Lenka Toukalkova, Vojtech Dotlacil, Katerina Greinerova, Anabela Cizkova, Rudolf Horvath, Jiri Bronsky, Jaroslav Havlik, Ondrej Hradsky, Ondrej Cinek

**Affiliations:** Department of Medical Microbiology, 2nd Faculty of Medicine, Charles University and Motol University Hospital, Prague, Czechia; Department of Paediatrics, 2nd Faculty of Medicine, Charles University and Motol University Hospital, Prague, Czechia; Department of Food Science, Faculty of Agrobiology, Food and Natural Resources, Czech Univesity of Life Sciences, Prague, Czechia; Department of Paediatrics, 2nd Faculty of Medicine, Charles University and Motol University Hospital, Prague, Czechia; Department of Paediatrics, 2nd Faculty of Medicine, Charles University and Motol University Hospital, Prague, Czechia; Department of Paediatrics, 2nd Faculty of Medicine, Charles University and Motol University Hospital, Prague, Czechia; Department of Pediatric and Adult Rheumatology, Motol University Hospital, Prague, Czechia; Department of Pediatric and Adult Rheumatology, Motol University Hospital, Prague, Czechia; Department of Paediatrics, Faculty of Medicine in Pilsen, Charles University and University Hospital Pilsen, Czechia; Department of Paediatrics, Faculty of Medicine, Palacky University Olomouc and University Hospital Olomouc, Czechia; Department of Paediatrics, 1st Faculty of Medicine, Charles University and Thomayer University Hospital, Prague, Czechia; Department of Paediatrics, 1st Faculty of Medicine, Charles University and Thomayer University Hospital, Prague, Czechia; Department of Paediatrics, Tomas Bata Hospital, Zlin, Czechia; Department of Paediatric Surgery, 2nd Faculty of Medicine, Charles University and Motol University Hospital, Prague, Czechia; Department of Paediatrics, Masaryk Hospital, Usti nad Labem, Czechia; Synlab Czech, Inc., Prague, Czechia; Department of Pediatric and Adult Rheumatology, Motol University Hospital, Prague, Czechia; Department of Paediatrics, 2nd Faculty of Medicine, Charles University and Motol University Hospital, Prague, Czechia; Department of Food Science, Faculty of Agrobiology, Food and Natural Resources, Czech Univesity of Life Sciences, Prague, Czechia; Department of Paediatrics, 2nd Faculty of Medicine, Charles University and Motol University Hospital, Prague, Czechia; Department of Medical Microbiology, 2nd Faculty of Medicine, Charles University and Motol University Hospital, Prague, Czechia; Department of Paediatrics, 2nd Faculty of Medicine, Charles University and Motol University Hospital, Prague, Czechia

**Keywords:** IBD, Crohn’s disease, anti-TNF, microbiome, metabolomics, children

## Abstract

**Background and Aims:**

Treatment with anti-tumour necrosis factor α antibodies [anti-TNF] changes the dysbiotic faecal bacteriome in Crohn’s disease [CD]. However, it is not known whether these changes are due to decreasing mucosal inflammatory activity or whether similar bacteriome reactions might be observed in gut-healthy subjects. Therefore, we explored changes in the faecal bacteriome and metabolome upon anti-TNF administration [and therapeutic response] in children with CD and contrasted those to anti-TNF-treated children with juvenile idiopathic arthritis [JIA].

**Methods:**

Faecal samples collected longitudinally before and during anti-TNF therapy were analysed with regard to the bacteriome by massively parallel sequencing of the 16S rDNA [V4 region] and the faecal metabolome by ^1^H nuclear magnetic resonance imaging. The response to treatment by mucosal healing was assessed by the MINI index at 3 months after the treatment started. We also tested several representative gut bacterial strains for *in vitro* growth inhibition by infliximab.

**Results:**

We analysed 530 stool samples from 121 children [CD 54, JIA 18, healthy 49]. Bacterial community composition changed on anti-TNF in CD: three members of the class Clostridia increased on anti-TNF, whereas the class Bacteroidia decreased. Among faecal metabolites, glucose and glycerol increased, whereas isoleucine and uracil decreased. Some of these changes differed by treatment response [mucosal healing] after anti-TNF. No significant changes in the bacteriome or metabolome were noted upon anti-TNF in JIA. Bacterial growth was not affected by infliximab in a disc diffusion test.

**Conclusions:**

Our findings suggest that gut mucosal healing is responsible for the bacteriome and metabolome changes observed in CD, rather than any general effect of anti-TNF.

## Graphical Abstract

**Graphical Abstract:**
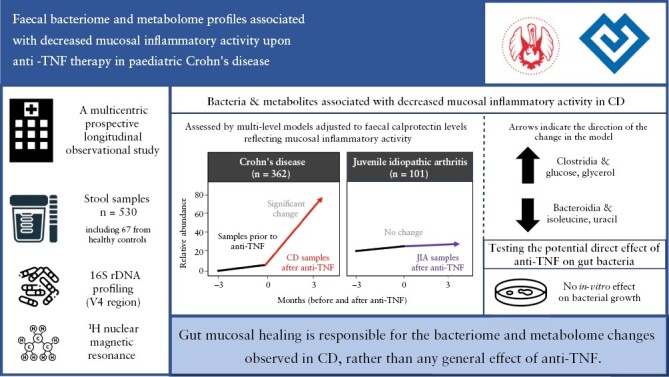

## 1. Introduction

Crohn’s disease [CD] is an inflammatory bowel disease [IBD] characterized by chronic inflammation of any part of the gastrointestinal tract with a progressive and destructive course; its incidence is increasing worldwide.^1,2^ Its aetiology remains unclear, with a strong contribution from the distorted microbiota.^3–13^ The disease is incurable, with a multitude of therapeutic strategies available: in the last decade, tumour necrosis factor alpha blockers [anti-TNF] have been established as easily available and highly effective treatments.^14^

The body of knowledge on the effect of anti-TNF on the faecal microbiome and metabolome is limited. One of the first studies by Wang *et al*. in a population of 11 children with CD reported that anti-TNF blockade in CD is associated with an increased diversity and a shift towards the composition seen in healthy controls. Among others, they observed a significant decrease in the relative abundances of inflammation-promoting Enterobacteriaceae but without a significant increase in gut health-promoting taxa, such as short-chain fatty acid [SCFA] producers.^15^ A subsequent investigation by the same team studied a population of 29 children with CD, of whom 18 received anti-TNF and 11 responded.^16^ The authors described an enrichment in several SCFA producers following anti-TNF treatment and showed that infliximab responders were characterized by higher abundances at baseline of some non-SCFA-producing genera [*Methylobacterium*, *Sphingomonas*, *Staphylococcus* and *Streptococcus*]. Additionally, they reported a shift in the quantity of several amino acids as a potential marker of anti-TNF response.^16^

Several recent studies aimed to predict the response to anti-TNF [namely infliximab] in IBD using the microbiome composition. Ventin-Holmberg *et al*. reported a lower abundance of SCFA producers at baseline as a marker of non-response to anti-TNF in adults.^17^ In a subsequent study in children, the response to anti-TNF was associated with several significant differences in bacterial abundance at baseline [namely, classes Clostridia and Bacilli vs class Gammaproteobacteria] and *Ruminococcus* [a known SCFA producer] combined with baseline calprotectin levels was a relatively good predictor of the response.^18^ Höyhtyä *et al*. studied the baseline composition of the bacteriome in children and young adults—this was done based on absolute and relative abundances. They found modest differences in absolute abundances [more Bifidobacteriales and less Actinomycetales in the remission group]; however, the relative abundances on the order level were reported only in Bifidobacteriales.^19^ The above study by Wang *et al*. also reported several borderline-significant associations of baseline bacterial abundance with the response to anti-TNF.^16^

Thus, there is compelling evidence that the gut microbiome of CD patients is altered upon anti-TNF therapy; however, it has not been established whether these microbiome shifts are due to decreasing mucosal inflammatory activity or whether anti-TNF may exert additional effects independent of the inflammatory activity and mucosal healing. A microbe specifically changing upon administering anti-TNF in CD but not reacting to anti-TNF in an extraintestinal diagnosis could indicate decreasing mucosal inflammatory activity. Therefore, in this study, we explored juvenile idiopathic arthritis [JIA] as such an extraintestinal paediatric diagnosis also treated with anti-TNF.

The aims of the present study were: [1] to characterize changes in the gut microbiome and metabolome associated with anti-TNF treatment, specifically in CD, but not in gut-healthy subjects with JIA—such changes would indicate decreasing mucosal inflammatory activity rather than a general effect of anti-TNF on immunity and the bacteriome; [2] to assess whether such bacteriome changes in CD differ by the indicators of mucosal healing 3 months after the start of anti-TNF therapy; and [3] to assess whether common anaerobic human gut bacteria are susceptible *in vitro* to the anti-TNF drug infliximab applied alone or with serum complement—lack of such susceptibility would argue against a direct anti-TNF effect on bacterial flora.

## 2. Methods

### 2.1. Study cohort

Children diagnosed with CD or JIA were invited to participate in a multicentre prospective longitudinal observational study performed at the Departments of Paediatrics, Adult and Paediatric Rheumatology and Paediatric Surgery, Motol University Hospital, Prague, and Departments of Paediatrics at University Hospital Pilsen, University Hospital Olomouc, Thomayer University Hospital, Prague, Tomas Bata Regional Hospital, Zlin, and Masaryk Hospital, Usti nad Labem, all Czechia. The study was carried out between July 1, 2019 and February 25, 2022. Participants could enter the study at two-time points: either at diagnosis or at indication for anti-TNF. The inclusion criteria were age between 3 and 17 years [3–16 for JIA patients] at the study entry, diagnosis of CD [based on Porto criteria]^20^ or JIA [based on ILAR criteria],^21^ and anti-TNF-naive state. Exclusion criteria in CD were ileocaecal stricture or co-occurrence of primary sclerosing cholangitis. In JIA, exclusion criteria were co-occurrence of diagnosed IBD or chronic diarrhoea with faecal calprotectin over 250 µg/g.^22,23^ Stool samples were collected regularly once per month from entering the study until 3 months [between the 12^th^ and 14^th^ weeks] after commencing the therapy with anti-TNF or February 2022, whichever occurred first [the study scheme is shown in Supplementary Figure 1].

In addition, control faecal samples were obtained from healthy controls recruited among [1] children hospitalized at the same facility for benign surgeries without a need for antimicrobial pretreatment [e.g. hernia or circumcision] and [2] young adults [under 21 years of age] recruited as possible donors in another ongoing study with faecal microbiota transplantation in patients with irritable bowel syndrome registered at clinicaltrials.gov under NCT04899869.^24^

Written informed consent was provided by the patient’s parents or guardians. The study was approved by the Ethics Committee of the Motol University Hospital on February 27, 2019 [EK-100/19]. The STROBE checklist was used for reporting the results of the study.^25^

### 2.2 Treatment with anti-TNFα antibodies

The anti-TNF therapy in CD was indicated either as a part of the step-up strategy or as a first-line therapy of the fistulizing perianal disease.^26^ Indication was always based on clinical criteria, and laboratory and endoscopic findings, independently of the present observational study. As anti-TNF therapy, treatment with infliximab or adalimumab was performed according to current guidelines.^26,27^ Infliximab was administered via an intravenous infusion of 5 mg/kg at weeks 0, 2, and 6, followed by maintenance intravenous infusions every 4–8 weeks; adalimumab was administered via subcutaneous injection at weeks 0, 2, and 4 in a scheme of 80–40–20 mg [up to 40 kg in weight] or 160–80–40 mg [above 40 kg in weight] followed by maintenance therapy with 25 mg/m^2^ up to 40 mg every 2 weeks.

In JIA, only adalimumab was used as anti-TNF therapy. It was indicated after primary or secondary failure of disease-modifying antirheumatic drugs [usually methotrexate] according to European Alliance of Associations for Rheumatology [EULAR] recommendations.^28^ Adalimumab was administered via subcutaneous injection every other week at a dose of 20 mg [up to 30 kg in weight] or 40 mg [above 30 kg in weight] without any induction scheme, as recommended by EULAR.^28^

### 2.3. Stool sample collection, faecal calprotectin analysis

Stool samples were collected using self-administered kits at the subject’s home [or rarely during hospitalization], placed in a pre-frozen transport container and stored in a home freezer until transportation to the clinic. At the clinic, samples were stored at −20°C and transported to a deep freezer [−80°C] shortly afterwards. Faecal calprotectin levels were measured in an accredited laboratory [ISO 9001:2015; Supplementary Methods, section 1] and served to indirectly assess mucosal inflammatory activity within every sample.

### 2.4. Sequencing of 16S rDNA and bioinformatic analysis of the bacteriome

The stool samples were thawed on ice, and DNA was extracted from 50–100 mg via a DNeasy PowerSoil Kit according to the manufacturer’s instructions on a QiaCube instrument [both Qiagen]. The quantity of bacterial DNA was then assessed using real-time PCR with primers and a hydrolysis probe targeting the V4 region of the 16S rRNA gene.

Bacteriome profiling was performed by sequencing the V4 region of the bacterial 16S rRNA gene according to Kozich *et al*.^29^ Bioinformatic processing was based on the DADA2 pipeline [version 1.22] as advised by the software authors.^30^ The procedures are described in detail in Supplementary Methods [sections 2.1 and 2.2].

### 2.5. Statistical analysis of the bacterial 16S rDNA profiles

#### 2.5.1. Alpha diversity of the bacteriome

Alpha [within-sample] diversity was assessed from the unfiltered rarefied dataset using the observed species counts, and Chao1, Shannon and Simpson indices. Multi-level models were built to characterize the changes in alpha diversity during observation of the subjects, with a random intercept for the subject and time as one of the predictors. The other tested predictors were the diagnosis, the therapy with anti-TNF during the collection of samples, faecal calprotectin levels, and their meaningful interaction.

After assessment of alpha diversity, we eliminated amplicon sequence variants [ASVs] without assigned phyla, those with extremely rare phyla likely to be only passive travellers through the gut, and taxa outside the kingdom Bacteria. Then *phyloseq* objects were created by taxonomic agglomeration at several taxonomic levels: phylum, class, order, family, genus, and the fine-grained level of ASV. Finally, rare taxonomic units were eliminated, defined as those not present in at least two samples at more than 0.1% of reads.

#### 2.5.2. Faecal calprotectin

Calprotectin concentrations were analysed using multi-level models similar to those employed in the alpha diversity analyses [i.e. with a random intercept for the subject and time as one of the predictors—other predictors were the diagnosis and whether the patient had anti-TNF therapy]. The dependent variable was log-transformed calprotectin concentration.

#### 2.5.3. Exploration of the community patterns

The bacteriome communities were first explored by Hellinger transformation-based principal component analysis [tb-PCA] ordination. Influential taxa in the ordination were found by inspection of the loadings and their formal testing using the *envfit* function. Associations between diagnosis, anti-TNF therapy, time since onset, alpha diversity measures and calprotectin levels were tested in principal component regression analysis models.

#### 2.5.4. Changes in abundance of taxa on anti-TNF therapy

The effect of anti-TNF therapy was assessed using multi-level models constructed similarly as for the tests of alpha diversity. The models included random intercepts for individuals, while the fixed effects were the diagnosis, time since the start of observation, faecal calprotectin levels, whether the sample was taken while on anti-TNF, and meaningful two-way interactions of the terms. Taxa with a convincingly significant association with anti-TNF therapy [raw *p* < 0.002] or with a strong dependence on time were further inspected by plotting the abundances over time by diagnosis and anti-TNF therapy. At each taxonomic level, the *p*-values for the anti-TNF terms were corrected using Bonferroni correction for the count of meaningfully testable taxa, i.e. those taxa whose abundance exceeded 0.1% [10 reads per 10 000] in at least 3% of studied samples.

#### 2.5.5. The bacteriome and response to anti-TNF therapy by mucosal healing

We analysed the associations of the gut bacteriome community with indicators of mucosal healing defined by using treatment effect data. The MINI index^31^ was assessed in CD patients as a proxy for mucosal healing at 3 months since the start of the anti-TNF treatment [i.e. between the 12^th^ and 14^th^ weeks]. A score <8 was classified as mucosal healing after anti-TNF treatment, whereas a score of ≥8 indicated inflammatory activity in the mucosa.

Multi-level models with random intercept for subjects were then used to test whether the change in taxa abundance upon anti-TNF therapy differed by the response to this therapy and whether any appreciable differences were detectable already at baseline. For this analysis, baseline was defined as available stool samples collected up to 3 months before the first administration of anti-TNF.

Data from stool bacteriome profiling and metabolome profiling were combined to investigate possible associations [N-data integration] using multiblock sparse projection to latent structures – discriminant analysis, as implemented in the MixOmics R package 32.

### 2.6. Stool metabolome profiling

An aliquot of stool [200 mg] was processed [detailed in Supplementary Methods, section 3.1] to obtain a final supernatant used for nuclear magnetic resonance [NMR] analysis. ^1^H NMR spectra were recorded on a 500-MHz Bruker Avance III spectrometer using a standard Bruker noesypr1d [90°-t1-90°-dmix-90°-FID] sequence as previously described by Jaimes *et al*.^32^ Spectral intensities of the ^1^H NMR spectra were pre-processed with an in-house built script under MATLAB R2020a [The MathWorks] consisting of multipoint baseline correction in user-defined segments, ensuring identical pre-processing for all the spectra (detailed in Supplementary Methods [section 3.2]). Spectra were reduced to predefined spectral bins, each bin representing a spin system or a part of a spin system that was ideally pure, distinct, and quantitative—in most cases, one bin for each metabolite. Ranges for the bins were chosen after the previous annotation of a subset of spectra in the software Chenomx version 8.6, using the built-in spectral library and our in-house database,^32^ as shown in Supplementary Table—Metabolites [sheet RawData]. The bins represented 57 unique annotated compounds, which were corrected for dilution factor using probabilistic quotient normalization. Normalized data given are in Supplementary Table—Metabolites [sheet ‘PQN-TSP normalised’].

Exploration of metabolome profiles used dimensionality reduction by principal component analysis [PCA] of the normalized and scaled metabolite levels. Multi-level models identical to those used above for bacteriome alpha diversity and taxon abundance were then used to test [1] the sample scores in the first four principal components [PCs], [2] individual metabolites, [3] the sum of SCFAs and their ratios to branched-chain fatty acids [BCFAs].

### 2.7. *In vitro* bacterial cultures with anti-TNF antibody

To test an unlikely direct effect of anti-TNF on the gut bacteria, we designed a simple *in vitro* disc diffusion assay. Isolates of bacteria commonly observed in the gut of healthy children were inoculated on Schaedler agar. Then, discs soaked with 20 µL of four different infliximab concentrations [increasing from 5 to 20 µg/mL] were applied on top of the inoculated agar and cultivated overnight under anaerobic conditions. These were used alone in one experiment and as a double-disc synergy test in another experiment—here, another disc was soaked with 20 µL of serum complement [concentration of 50 µg/mL] and placed near the infliximab disc to allow synergy between the antibody and complement action. Only Gram-negative bacteria were tested because complement activation is known to damage their cell walls, unlike Gram-positive bacteria, whose thick peptidoglycan layer makes them resistant to complement membrane attack complex.^33^ The infliximab concentration was selected to mimic or exceed theoretical serum concentrations after infusion; the complement concentration reflected routine laboratory settings for purposes of the complement-fixation test. Plates were inspected and photographed after overnight anaerobic incubation for inhibition zones or growth enhancement.

## 3. Results

### 3.1. Subjects and their samples

A total of 121 subjects [CD 54, JIA 18, healthy controls 49] met the inclusion criteria, their parents signed the informed consent, and thus they entered the study. The basic characteristics of the studied subjects are shown in Table 1 and Supplementary Table 1. Of 54 analysed subjects with CD, 37 progressed to the need for anti-TNF therapy, whereas 17 remained on other therapeutic modalities. Among children with JIA, 14 received anti-TNF therapy during the observation period, whereas four remained on other treatments.

**Table 1.** Characteristics of studied subjects and their samples.

	Crohn’s disease [*n* = 54]			Juvenile idiopathic arthritis [*n* = 18]			Healthy control [*n* = 49]
Status	Total	Received anti-TNF	Without anti-TNF	Total	Received anti-TNF	Without anti-TNF	
No. of subjects	54	37	17	18	14	4	49
Age at diagnosis, median in years [IQR]	12.6 [9.2–15.0]	12.9 [9.1–15.0]	12.5 [10.2–14.9]	8.6 [3.8–10.7]	8.6 [3.8–10.8]	8.8 [6.8–10.0]	–
Female sex, *n* [%]	22 [41%]	15 [41%]	7 [41%]	11	9 [64%]	2 [50%]	20 [41%]
Caucasian ethnicity, *n* [%]	52 [96%]	35 [95%]	17 [100%]	18	14 [100%]	4 [100%]	49
Family history of IBD, *n* [%]	11 [20%]	9 [24%]	2 [12%]	**–**	–	–	–
No. of samples per patient, median [IQR]	6.0 [5.0–8.0]	6.0 [5.0–8.0]	6.0 [3.0–9.0]	6.0 [4.0–6.75]	6.0 [5.0–6.75]	4.0 [3.75–5.75]	1.0 [1.0–1.0]
Faecal calprotectin concentration [µg/g]; median [IQR] of patient means	1158 [219–1862]	1424 [655–1988]	219 [52–1807]	20 [10–55]	24 [10–56]	11 [6.4–16]	11 [7–21]
**Treatment regimes** ^a^							
Immunomodulator monotherapy [azathioprine/methotrexate]	13 [24%]	–	13 [76%]	3 [17%]	–	3 [75%]	–
Anti-TNFα monotherapy^b^	19 [35%]	19 [51%]	–	4 [22%]	4 [29%]	–	–
Combined immunomodulatory + anti-TNFα	18 [33%]	18 [49%]	–	10 [56%]	10 [71%]	–	–
Other treatment^c^	4 [7%]	–	4 [24%]	1 [6%]	–	1 [25%]	

^a^Distribution of immunomodulatory agents: in CD 31/54 [57%] were treated with immunomodulatory agents [azathioprine 25, methotrexate 6]; in JIA 13/18 [72%] were immunomodulatory agents [all methotrexate].

^b^Distribution of anti-TNF agents in CD: infliximab 18/37 [49%]; adalimumab 19/37 [51%]; in JIA, all subjects received adalimumab as anti-TNF.

^c^In CD, three out of the four subjects provided samples only before the diagnosis and any treatment of CD; one subject was treated without immunomodulatory agents with supplementary enteral nutrition only; in JIA, this one patient was treated with non-steroid anti-inflammatory drugs only.

IQR = interquartile range.

**Figure 1.**
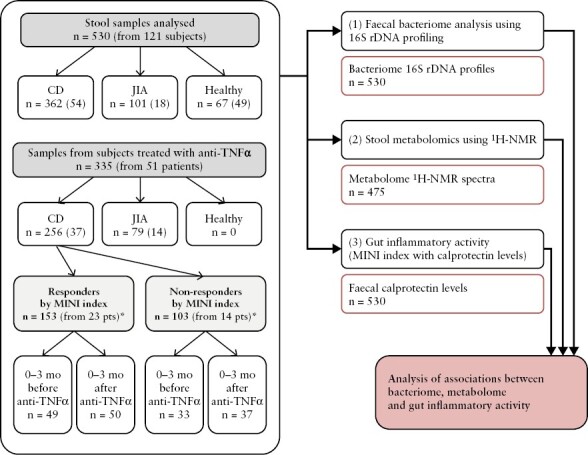
Flowchart of the study. *n* are counts of analysed samples [in parentheses are the numbers of subjects from whom the samples were obtained]. *Count also includes samples taken outside the 6-month window encompassing the start of anti-TNF.

Altogether, we collected and analysed 530 stool samples: 362 samples were from 54 patients with CD [203 samples taken while the patient was off anti-TNF, and 159 on anti-TNF], 101 samples were from 18 patients with JIA [63 samples before therapy and 38 on anti-TNF], and 67 samples were from 49 control subjects. Associations between the bacteriome or metabolome composition and the response of patients with CD to anti-TNF, assessed by the MINI index, were studied using subsets of samples taken up to 3 months before the first anti-TNF dose vs those taken up to 3 months after the initiation this therapy: there were 99 such stool samples from 23 CD patients who responded [49 before, 50 after the initiation of therapy], and 70 samples from 14 CD patients who did not respond [33 before, 37 after the initiation of therapy]. A flow chart of the study is shown in Figure 1; stool samples and the dynamics of their collection over time in each subject are shown in Supplementary Figure 2.

### 3.2. Mucosal inflammatory activity

In JIA, calprotectin levels were not different from controls [*p* = 0.16], and no association was noted for calprotectin with time or with anti-TNF therapy.

In patients with CD, faecal calprotectin levels were expectedly higher than in controls [~30-fold, *p* < 10^−16^] as well as than in JIA [20-fold, *p* < 10^−12^]. Those CD patients who eventually proceeded to anti-TNF therapy had 8.5-fold higher mean calprotectin levels over the whole study period as compared to those who remained on other therapies. When anti-TNF therapy was administered to CD patients, their calprotectin levels decreased by a mean of 67% as compared to the period off-therapy [*p* = 1.2 × 10^−7^; Supplementary Figure 3]. Analysis of calprotectin levels in stools from CD patients was then restricted to 3 months before commencing anti-TNF therapy and the following first 3 months of this therapy, with a multi-level model having a random intercept for subject and interaction terms for time and therapy: the results remained virtually unchanged, with the mean decrease in calprotectin with anti-TNF being 70% [*p* = 0.0015], and no appreciable independent effect of time.

In patients with CD receiving anti-TNF, 23 [62%] achieved mucosal healing as defined by MINI index <8, and 14 subjects [38%] did not.

### 3.3. Bacteriome profiles and anti-TNF therapy

#### 3.3.1. Alpha diversity

The alpha [intra-sample] diversity of samples from CD patients was substantially lower than in those from control subjects [*p* < 10^−5^ in Observed species, Chao1, and Shannon indices, and *p* = 0.008 for Simpson index in mixed-effect models with time, diagnosis, log-transformed calprotectin levels and anti-TNF therapy as fixed effects, and subject as a random effect; alpha diversity index was the dependent variable]. Compared to JIA, samples from patients with CD had lower values of Shannon [*p* = 0.001] and Simpson indices [*p* = 0.020], but no significant difference was noted in Chao1 and Observed species indices [Supplementary Figure 4].

In patients with CD, all alpha diversity indices increased with time during the longitudinal observation [*p*-values between 0.003 and <10^−6^ for the above indices]. The effect of time as a predictor is shown in Supplementary Figure 5. Some but not all indices showed suggestive negative associations with log-transformed calprotectin levels [*p* = 0.042 for the observed count of species, *p* = 0.039 for Chao1, *p* = 0.094 for Shannon, *p* = 0.15 for Simpson]. Ongoing anti-TNFα therapy was weakly independently associated with an increase in the Simpson index [*p* = 0.024] but not with the other tested indices. No significant association of the alpha diversity indices was noted with mucosal healing [assessed by MINI index 3 months after commencing anti-TNF] among CD patients on anti-TNF therapy.

No such differences or trends were observable in JIA: here, none of the alpha diversity indices changed with time [Supplementary Figure 5]; calprotectin levels were low, not appreciably different from control subjects, and were not associated with alpha diversity.

#### 3.3.2. Bacteriome community exploration

**Figure 2.**
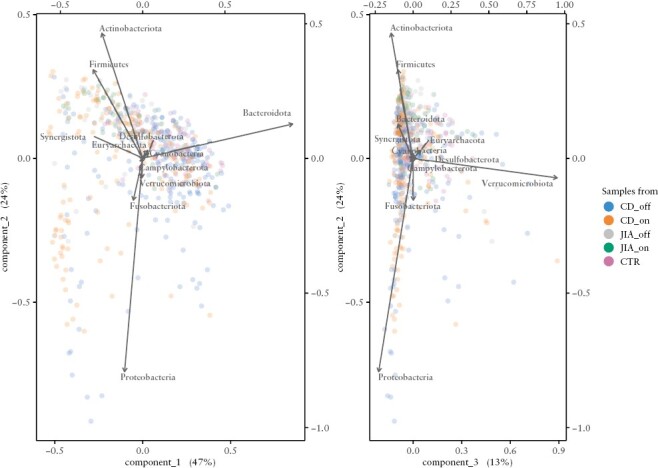
Bacteriome composition with the PCA showing the vectors for phyla. Samples from CD patients off therapy were driven mostly by the phylum Bacteroidota, including genera with colitis-inducing potential,^67^ which are found more often in CD patients thanin healthy individuals,^40^ and by the phylum Proteobacteria, driven mostly by genera associated with dysbiosis in CD patients.^37^ The samples of CD on therapy were closer to the clusters of JIA patients, and healthy controls, driven mostly by the phylum Firmictues with genera known to be associated with better gut health.^37,40^ The change in the bacteriome composition can be explained by both the gut health status and the bacteriome’s alpha-diversity. Put simply, the more Bacteroidota [more negative on axis 1], the lower the alpha diversity, and similarly, the more Proteobacteria [the more negative on axis 2], the lower the diversity and the worse gut health.

In PCA ordination of the Hellinger-transformed abundance table at the phylum level, the first three dimensions were needed to explain >80% of the variance [47, 24, and 12% of the total variance; Figure 2]. PC1 captured the balance between Bacteroidota on one side and Firmicutes with Actinobacteriota on the other side. PC2 was driven by Proteobacteria and Fusobacteriota to negative values, counterbalanced by Actinobacteriota and Firmicutes. PC3 was driven by Verrucomicrobiota [for the abundance gradients of relevant phyla, see Supplementary Figure 6A and B]. Samples from control subjects and patients with JIA, as well as many samples from CD patients, were placed along the first PCs forming a cloud-like cluster. This largest variance component reflected the ratio between Bacteroidetes [right, with positive PC1 values] and Firmicutes with Actinobacteriota [left, negative PC1 values; Figure 2, left panel]. Numerous samples from CD patients and a few from JIA patients departed from the main cluster along the PC2 axis to negative values [i.e. towards an increasing abundance of Proteobacteria and/or Fusobacteriota]. Consequently, a significantly higher dispersion of the CD samples was noted compared to samples from control subjects or JIA patients [*p* = 0.001].

#### 3.3.3. Testing associations of the bacteriome community composition using principal component regression

We explored putative associations of the PCA scores by multi-level models adjusted for known confounders. Community composition of the bacteriome was associated with alpha diversity: significant direct associations were noted for the first two PCs with all indices—i.e. the higher the score on the axis [towards Bacteroidota on PC1 and towards Firmicutes with Actinobacteriota on PC2], the higher the alpha diversity. Diversity scores were particularly low in samples with abundant Proteobacteria and Fusobacteria [Supplementary Figure 6C]. In addition, Simpson and Shannon indices were inversely associated [*p* = 0.001] with PC3 [i.e. these two indices decreased with increasing abundance of Verrucomicrobiota].

Faecal calprotectin in a stool sample was inversely associated with PC2 [*p* = 1.3 × 10^−5^] and PC3 [*p* = 0.001] of its bacteriome community composition, i.e. calprotectin was higher with increasing Proteobacteria [*p* < 10^−6^] and/or Fusobacteriota [*p* = 0.002] abundnace, but marginally lower with increasing Verrucomicrobiota [*p* = 0.042] [Supplementary Figure 6D]. The association signal originated solely from subjects with CD. No such association was noted for PC1.

Anti-TNF therapy was associated with the faecal bacteriome community composition in patients with CD. There was an inverse association of anti-TNF therapy with PC1 [*p* = 0.00017] and PC2 [*p* = 0.0012, both in multi-level models adjusted for calprotectin]. Samples taken after anti-TNF therapy shifted from Bacteroidota towards an increased abundance of Firmicutes or Actinobacteriota. In JIA, neither of the PCs was associated with anti-TNF therapy.

Subjects with mucosal healing differed from those without in the reaction of the community composition to anti-TNF therapy. PC1 did not differ at baseline, but only samples from subjects without mucosal healing changed upon this treatment [towards negative values, *p* = 3 × 10^−5^]. PC2 differed already at baseline [patients with mucosal healing being higher, *p* = 0.011]; patients reacted to therapy with an increase in PC2 [*p* = 0.0073], regardless of the response by mucosal healing and with no appreciable interaction between the pace of increase and the response. In PC3, no baseline difference nor differential change was noted among categories of mucosal healing.

#### 3.3.4. Abundance of individual bacterial taxa changes upon anti-TNFα therapy

**Figure 3.**
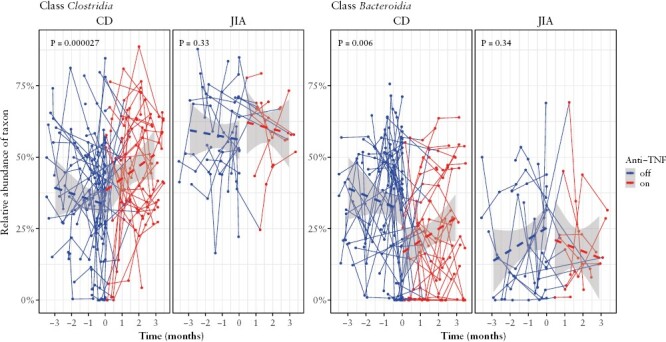
Relative abundance of classes Clostridia and Bacteroidia with time. Diagnosis: CD = Crohn’s disease, JIA = juvenile idiopathic arthritis. Colours by therapy of anti-TNFα: red = stool sample taken while on anti-TNF; blue = stool sample taken when no anti-TNF was administered. The *p*-value is corrected for the number of tested taxa using multi-level modelling.

We searched for taxa that were significantly changed in connection with the administration of anti-TNF therapy in CD but did not change upon TNFα blockade in JIA. No taxon changed significantly upon anti-TNF therapy in JIA [the smallest corrected *p* = 0.38]. In CD, significant changes upon anti-TNFα [corrected *p* < 0.05] were noted in two phyla, two classes, four families, and four genera [Table 2, Figure 3, Supplementary Table 2]. The phylum Bacteroidota, class Bacteroidia, order Bacteroidales decreased by 8.1 percentage points upon therapy [corrected *p* = 0.0061]. In Firmicutes, an increase was noted for the whole phylum, as well as in the class Clostridia and orders Peptostreptococcales and Lachnospirales. At the genus level, *Alistipes* was the only one which decreased upon anti-TNF in CD, whereas *Intestinibacter*, *Ruminococcus*, and *Flavonifractor* increased.

**Table 2.** Bacterial taxa whose quantity changed significantly with anti-TNF therapy in CD

	IBD	The change with anti-TNF therapy in patients with IBD		JIA	The change with anti-TNF therapy in patients with JIA		Notes
Bacterial taxon	Mean taxon abundance in IBD patients [% of total reads]	Mean abundance change in therapy [percentage points]	*p*-value: raw [corrected for number of tested taxa]	Mean taxon abundance in JIA patients [% of total reads]	Mean abundance change on therapy [percentage points]	Raw *p*-value [1]	
p. Bacteroidota └─ c. Bacteroidia └─ o. Bacteroidales	29%	−8.1	6.2 × 10^−4^ [0.0061]	22%	+4.1	0.34, NS	[2]
└─ f. Rikenellaceae │	2.6%	−1.9	2.3 × 10^−5^ [9.2 × 10^−4^]	2.4%	+0.68	0.38, NS	
└─ g. *Alistipes*	2.7%	−2.0	2.1 × 10^−5^ [0.0024]	2.4%	+0.71	0.37, NS	
p. Firmicutes	54%	+9.7	1.7 × 10^−5^ [1.7 × 10^−4^]	64%	−4.8	0.19, NS	
└─ c. Clostridia │	43%	+11	2.0 × 10^−6^ [2.7 × 10^−5^]	58%	−3.6	0.33, NS	
│─ o. Peptostreptococcales │	2.0%	+1.4	2.2 × 10^−4^ [0.0055]	1.8%	+0.29	0.53, NS	[3]
│ └─ f. Peptostreptococcaceae │	1.7%	+1.1	4.3 × 10^−4^ [0.017]	1.7%	+0.35	0.44, NS	
│ └─ g. *Intestinibacter* │	0.74%	+0.75	1.6 × 10^−5^ [0.0019]	0.56%	+0.13	0.47, NS	
│─ o. Lachnospirales │	26%	+9.4	1.5 × 10^−7^ [3.7 × 10^−6^]	30%	−4.3	0.12, NS	
│ └─ f. Lachnospiraceae │	26%	+9.4	1.6 × 10^−7^ [6.4 × 10^−6^]	31%	−4.1	0.14, NS	
│ └─ g. *Ruminococcus* │	2.3%	+2.7	1.4 × 10^−4^ [0.015]	0.2%	0.0	0.94, NS	[4]
└─ ..... g. *Flavonifractor*	0.4%	+0.38	3.7 × 10^−4^ [0.042]	0.2%	−0.05	0.32, NS	[5]

[1] Neither of the tested taxa yielded a corrected *p*-value of the mean change after anti-TNF in JIA lower than 0.05. There were 13 taxa having a raw uncorrected *p*-value <0.05 in this test. Neither of them had a mean abundance >1% in either CD or JIA, and all these spurious *p*-values were caused by one or a few outliers, as confirmed by inspection of the abundance graphs.

[2] The three taxonomic categories completely overlapped in our human faecal dataset. There were no Bacteroidia other than Bacteroidales and no Bacteroidota other than Bacteroidia.

[3] The order is named Peptostreptococcales–Tissierellales in the SILVA database v. 138.

[4] Classified as *R. gnavus* group by SILVA database v. 138.

[5] Genus *Flavonifractor*, class Clostridia, order Oscillospirales, family Oscillospiraceae [SILVA database v. 138].

The regression coefficient corresponds to a mean increase or decrease associated with the anti-TNF therapy. Here it is expressed as a change in percentage points, as the bacterial abundance enters the model as a relative count of reads per 10 000.

At each taxonomic level, those taxa were tested whose abundance exceeded 0.1% [10 reads per 10 000] in at least 3% of studied samples. This resulted in a Bonferroni correction for 115 tests at the genus level, 40 tests at the family level, 26 at the order level, 13 at the class level, and 10 at the phylum level.

NS, not significant; p., phylum; c., class; o., order; f., family; g., genus.

#### 3.3.5. The bacteriome changes in responders and non-responders to anti-TNF therapy

Some of the above listed changes in bacterial taxa differed by whether the subject clinically responded to the therapy or not. Those patients responding with mucosal healing [defined by the composite MINI index being <8 at 3 months after commencing anti-TNF therapy] had a less pronounced increase in Firmicutes as compared to non-responders [corrected *p* < 0.05; similarly to class Clostridia and order Lachnospiraceae], and a less pronounced decrease in the phylum Bacteroidota, its family Rikenellaceae and its genus *Alistipes* [corrected *p* < 0.05]. The pattern was rather complex. The differential response to anti-TNF therapy in Firmicutes is explainable by baseline differences: future responders had more Firmicutes at baseline [by 15 percentage points, *p* = 5.3 × 10^−3^], so the increase in responders was less pronounced [*p* of interaction 8.5 × 10^−4^]. In contrast, Bacteroidota, its family Rikenellaceae and its genus *Alistipes* did not differ appreciably at baseline [*p* = 0.72], and only non-responders reacted to anti-TNF with a decrease [*p* = 8.9 × 10^−4^].

Having detected this effect modification, we performed an unplanned secondary exploration of whether the abundance of any taxon at baseline predicted the future responder status. For baseline we used taxon abundances in samples collected during the 3 months immediately preceding the start of anti-TNF. Several suggestive association signals emerged: at the phylum level [apart from the above reported higher abundance of Firmicutes], future responders had a lower abundance of Proteobacteria [by 14 percentahe points, uncorrected *p* = 0.035], propagating from the level of class Gammaproteobacteria and order Enterobacterales with similar difference and *p* values, as well as to the second principal component of the bacteriome ordination [Supplementary Table 3].

#### 3.3.6. Disc diffusion testing potential direct effects of anti-TNF [infliximab] and/or serum complement on bacterial cells

We tested direct infliximab effects on six isolates of bacteria commonly observed in stool of healthy children: *Clostridium innocuum*, *Anaerococcus degeneri*, *Finegoldia magna*, *Prevotella nigrescens*, *Bacteroides fragilis*, and *Escherichia coli*. A disc-diffusion test with four different concentrations of infliximab assessed the potential effect of anti-TNF: *A. degeneri* had insufficient growth even after 48 h of incubation; *C. innocuum* and *F. magna* had a very discrete, <1-mm zone of irregular growth around some but not all discs with infliximab—this was deemed not to be consistent with susceptibility and concluded to be a mechanical effect or an effect of diluent diffusion; isolates of *P. nigrescens*, *B. fragilis*, and *E. coli* showed no visible zones around any of the infliximab discs. The Gram-negative bacteria *P. nigrescens*, *B. fragilis*, and *E. coli* were then subjected to a double-disc synergy test with complement, showing no visible inhibition zones around either infliximab or complement discs.

### 3.4. Metabolome profiling

Of 530 stool samples, 55 specimens mostly from control subjects had insufficient total quantity and were used up during processing for bacterial DNA extraction. The metabolome was thus analysed in 475 faecal specimens: 355 from patients with CD [194 before therapy and 161 after], 98 from patients with JIA [61 before therapy and 37 after], and 22 from control subjects. A total of 57 faecal metabolites were annotated, including primarily amino acids, SCFAs, BCFAs, other organic acids, carbohydrates, polyamines, choline metabolites, phenolic acids, nucleobase derivatives, and others.

#### 3.4.1. Exploratory analysis of the metabolome

In PCA of the log-transformed scaled metabolome data, the first four PCs explained 42% of the total variance [16, 12, 7.4% and 7.0%], and the contributions declined thereafter. Of these axes, an increase in PC2 score was significantly associated with anti-TNF therapy in CD [*p* = 1.6 × 10^−4^ in a multi-level model adjusted for time, diagnosis, and calprotectin levels], i.e. towards higher concentrations of glucose, fucose, and glycerol, and lower concentrations of 4-aminobutyrate, glutamate, isoleucine, leucine, and valine **[**Supplementary Figure 7].

The above association was due to an increase in PC2 score in non-responders to anti-TNF among CD patients [interaction of the terms for anti-TNF treatment and being a responder *p* = 0.010], whereas no appreciable difference was present at baseline before anti-TNF treatment between CD patients who subsequently responded and those who did not.

In JIA, administration of anti-TNF was not associated with changes in any of the metabolome PC scores [uncorrected *p* values for the first four PC scores being 0.36, 0.56, 0.80, and 0.97].

#### 3.4.2. Abundance of individual faecal metabolites upon anti-TNF therapy

The 57 annotated bins from the ^1^H NMR spectra were tested by multi-level models similar to the bacteriome analysis. Four of the investigated metabolites changed significantly with anti-TNF therapy in CD [corrected *p* < 0.05, Table 3]: glucose and glycerol increased during anti-TNF therapy, whereas isoleucine and uracil decreased [Supplementary Figure 8].

**Table 3.** Faecal metabolites whose quantity changed significantly with anti-TNF therapy in CD.

	The change with anti-TNF therapy in patients with CD		Statistics for these metabolites in patients with JIA^b^	
	Metabolite abundance change on therapy^a^	*p*-value: raw [corrected for the number of tested metabolites]	Metabolite abundance change on therapy^a^	Raw *p*-value
Isoleucine	Decrease	1.6 × 10^−4^ [0.004]	Increase	0.84, NS
Uracil	Decrease	9.6 × 10^−4^ [0.002]	Increase	0.94, NS
Glucose	Increase	0.002 [0.03]	Decrease	0.38, NS
Glycerol	Increase	6.17 × 10^−5^ [0.003]	Decrease	0.94, NS

^a^Coefficients are not reported as they reflect the absolute change in units of the area under the peak, which is meaningless to interpret.

^b^In JIA, none of 57 tested metabolites showed a significant change with anti-TNF: the lowest raw *p*-value was 0.027, corresponding to a corrected *p* of 0.74.

NS, not significant.

There were no indications that individual metabolites at baseline could predict future response to anti-TNF in CD patients [lowest uncorrected *p* value 0.035].

Anti-TNF therapy in JIA was associated with no metabolite change [lowest uncorrected *p* value 0.027, corrected 0.996].

#### 3.4.3. SCFAs upon anti-TNF therapy

Since SCFAs have been the subject of previous studies, we evaluated their estimated concentrations, the sum of their concentrations, and their ratios to BCFAs in a separate model: no appreciable association was noted for anti-TNF therapy [*p* = 0.44], calprotectin level [*p* = 0.55], or time [*p* = 0.12], and there was no difference between JIA and CD [*p* = 0.19]. Similarly, there was no appreciable association of anti-TNF therapy and the SCFA/BCFA ratio [data not shown]. No association with anti-TNF was found for individual SCFAs, either [in CD, all *p*_corr_ = 1.0].

### 3.5. Combined analysis of bacteriome and metabolome profiles

The datasets containing the bacteriomes [at the level of genus] and metabolomes were correlated, yielding correlation coefficients of 0.85, 0.77, and 0.68 for variates of the first components in partial least squares regression. The co-inertia plot is shown in Supplementary Figure 9. The permutation co-inertia test for the two tables was highly significant [*p* < 10^−4^ by coinertia.randest of the package *ade4* in R].

In an analysis we then explored whether N-data multiomic integration [bacteriomes + metabolomes of the same samples] might perform better than bacteriomes only in simplified machine learning models [random forest and sparse projection to latent structures – discriminant analysis]. No improvement in accuracy was noted [data not shown].

## 4. Discussion

Our study describes changes in the faecal bacteriome and metabolome upon anti-TNF therapy in CD. It identified several bacterial taxa and metabolites that changed with anti-TNF, as well as taxa associated with the response to this therapy. Importantly, no such effects of anti-TNF on the faecal bacteriome or metabolome were observed in patients with JIA, who all were gut-healthy. Thus, this difference among two diagnoses treated with anti-TNF indicates that the observed changes in the bacteriome and metabolome reflect a reduction in mucosal inflammation and are not a general property of any anti-TNF administration [e.g. through a modification of the immune response to colonic bacteria]. This notion may be further supported by the absence of direct effects of infliximab in our *in vitro* growth experiments on anaerobic bacterial cultures.

### 4.1. Changes in the faecal bacteriome upon anti-TNF administration

Gut dysbiosis, in which the composition of the faecal bacteriome is very different from that considered normal, is a common feature of an anti-TNF-naive bacteriome in CD.^34,35^ Its main signs are an overall decrease in diversity and a different composition compared to healthy subjects: a decrease in obligate anaerobes or bifidobacteria and an increase of facultative anaerobes [e.g. Enterobacteriaceae from Proteobacteria] or some Gram-negative anaerobes such as *Bacteroides*.^34,35^ Consistent with multiple studies on the bacteriome in IBD of heterogenous design and methodology,^3–13^ we also observed significant differences between CD and gut-healthy comparison groups [JIA and healthy controls] in both diversity and composition of the bacteriome. The alpha diversity in CD was remarkably reduced, gradually improving with time since entering the study. Interestingly, time remained its only strong predictor, with a weak contribution from calprotectin and none from anti-TNF. This suggests that the alpha diversity increase in time could be due to any type of therapy of CD, and not limited to anti-TNF. It is of note that a similar non-superiority of anti-TNF on alpha diversity course has been reported in relatively small longitudinal studies of Chinese children with IBD using stool^16^ or biopsy samples.^36^ The composition of the bacteriomes among children with CD was notably more diverse, with that od some samples being very different to compositions observed in children with JIA or in healthy controls. In particular, the samples of anti-TNF-naive CD patients were enriched in taxa among the phyla Proteobacteria and Fusobacteriota, a well-known sign of gut dysbiosis in CD, and which has been reported in multiple cross-sectional studies.^6,7,11,12,34,37^ Altogether, these results confirm the dysbiotic patterns in CD even from the perspective of our longitudinal stool sampling.

The effect of anti-TNF on the human microbiota has been studied previously, primarily due to the infectious complications caused by these agents, as inhibiting the TNFα pathway increases the risk of several infectious diseases [tuberculosis, listeriosis, aspergillosis, coccidioidomycosis, histoplasmosis, or salmonellosis].^38^ However, only a few studies have focused on changes following anti-TNF therapy in IBD. They reported a shift in towards a healthier composition , namely a decrease in Proteobacteria and/or increase of SCFA producers.^15,16,39^ In our study, the bacteriome community composition upon anti-TNF in CD shifted from Bacteroidota towards Firmicutes. Such a shift could be considered a change towards a healthier gut bacteriome, as Bacteroidota are reported to be enriched among IBD patients in multiple studies,^7,8,12,40^ whereas genera of the Firmicutes are associated with better gut health.^6,9–12,37,40^ Thus, regarding the bacteriome composition, our results from a longitudinally collected sample series are consistent with previous studies on the paediatric population.^15,16,39^

Testing of individual taxa for the change upon anti-TNF administration revealed no shifts in JIA, but identified several taxa changing in CD: *Alistipes* [class Bacteroidia] decreased in abundance after therapy with anti-TNF, whereas *Intestinibacter*, *Flavonifractor*, and *Ruminococcus* [all class Clostridia] increased. Notable were *Ruminococcus*, from the family Lachnospiraceae, and *Flavonifractor*, from the family Oscillospiraceae. Both bacteria are known producers of SCFAs, critical nutrients for the gut epithelium.^41,42^*Ruminococcus* has been reported to be more abundant in a healthy population when compared to IBD,^7,9^ and its increase has been associated with a response to anti-TNF therapy in CD.^15,16^ On the other hand, some species of *Ruminococcus* can produce pro-inflammatory molecules *in vitro*^43^ and are associated with IBD^44^; and SCFA producers from the family Lachnospiracecae were found to decrease in responders to anti-TNF, which even the authors commented as being unexpected.^17^*Flavonifractor* is a phylogenetically diverse genus formerly classified as *Eubacterium*.^45^ Its primary representative *F. plautii* [also known as *E. plautii*] is, with the other eubacteria, a well-recognized SCFA producer found in the healthy gut.^46^ Of note, its close relative, *E. rectale*, was reported to be significantly increased at baseline in children responding to anti-TNF in CD.^39^ A connection with CD has already been reported for the genus *Intestinibacter*^47^ from the family Peptostreptococcaceae; however, disease activity [remission vs active disease] and gut inflammation status was not considered. More interestingly, an increase in an unknown genus from the Peptostreptococcaceae was reported to be a sign of anti-TNF response in children with IBD.^17^ The role of *Alistipes*, belonging to the order Bacteroidales, remains unclear.^48^ However, its connection with worse gut health seems probable based on recent studies,^49,50^ and thus its decrease after therapy may be meaningful.

### 4.2. Response to anti-TNF therapy and gut bacteria

The reaction of the bacteriome differed between responders based on the MINI index and non-responders. The pattern was complex, also involving the baseline abundance of the microbe. Bacteroidota started at similar baseline levels in responders and non-responders, and only non-responders subsequently experienced an increase, possibly indicating deterioration of their bacteriome dysbiosis. In contrast, it appeared that Firmicutes in responders deviated less at baseline, and thus their increase was less pronounced. Differences at baseline among responders and non-responders were also noted in Proteobacteria, another candidate for dysbiosis. The above taxa are valid candidates for being causatively involved: Clostridia of Firmicutes are known SCFA producers, whereas Enterobacterales are known pro-inflammatory bacteria.^6^

Numerous previous studies have presented a heterogeneous spectrum of candidates for microbial predictors of response to biological therapies—both in children and in adults with IBD, on infliximab,^16,17,19,51^ vedolizumab,^10^ or a combination of agents.^52^ The existence of such predictors questions the secondary origin of such changes. Given that there is a microbial predictor and its abundance increase is due to a reduction in mucosal inflammation, a third factor should exist [a mediator, confounder, or effect modifier]. Such a factor could be, for example, a CD susceptibility gene such as the interleukin 23 receptor [*IL23R*] previously found to be associated with a decreased diversity and abundance of beneficial taxa [e.g. *Faecalibacterium*].^53^ However, studies of this relationship are currently lacking.

Although prokaryotes possess no receptors that could be blocked by anti-TNF therapy, and thus a direct effect of anti-TNF on bacterial cells is unlikely, it cannot be ruled out that the monoclonal antibody binds non-specifically to bacterial structures. Therefore, we designed a series of simple experiments by exposing commonly occurring gut anaerobes to anti-TNF [infliximab] alone or together with serum complement in Gram-negative bacteria. Neither the disc diffusion test with discs soaked with infliximab on culture plates nor the double disc synergy test with infliximab and serum complement showed appreciable growth inhibition or growth enhancement. Given that commensal growth is not affected *in vitro*, that the purified therapeutic antibody is unlikely to cross-react with bacterial structures, and that there is no TNF-like pathway in prokaryotes, we concluded that a direct effect is most unlikely.

Overall, in the context of current the literature, we suggest that the changes in bacteriome indicate a meaningful connection between the associated taxa and healthier gut bacteriome composition and, thus, a decrease in mucosal inflammatory activity. Furthermore, the lack of such observations in gut-healthy JIA and the resistance of bacterial cultures to infliximab together suggest that bacteriome changes after anti-TNF are probably mediated by improved gut health in CD.

### 4.3. Changes in the faecal metabolome

Four faecal metabolites changed in concentration significantly with anti-TNF in CD patients, but no appreciable changes were noted in JIA [Table 3]. A decrease in isoleucine [and other amino acids prior to correcting for multiple comparisons] was observed. Excessive amounts of bacterial proteolytic enzymes were previously reported in faeces and biopsy samples from patients with active IBD.^54^ Amino acids are also known to be enriched in stool samples of CD [or ulcerative colitis] patients during a disease flare compared to remission.^55,56^ Moreover, a decrease in multiple amino acids [but not isoleucine] after therapy was described in children with CD.^16^ Thus, the decrease in isoleucine seen in our study might be connected to mucosal healing or the establishment of bacterial homeostasis. Uracil, another metabolite showing a decrease, was previously shown to be actively secreted by *Escherichia coli* and *Pseudomonas aeruginosa*, playing a role in quorum sensing, biofilm formation, and pathogenicity.^54,57^ Purine and pyrimidine pathways were recently reported to be down-regulated with diet-induced remission.^58^

The observed increase in glucose [Table 3] may relate to an increased capacity of microbiota to ferment dietary non-digestible fermentable carbohydrates and, thus, a shift from proteolytic towards saccharolytic fermentation. Glycerol can be derived from dietary fats and is commonly used as a food additive. It escapes absorption in the small intestinal, undergoes bacterial catabolism in the colon, and its rate varies in different microbial communities. Shifts in *Lactobacillus*–*Enterococcus* communities were associated with changes in glycerol, and their abundance was positively associated with SCFA production and negatively with BCFAs in batch fermentation studies.^59,60^

In general, all the changes among significant metabolites support the overall picture of the shift from proteolytic towards saccharolytic fermentation,^61,62^ which is considered a healthier profile.^63^ Interestingly, SCFAs are generally considered anti-inflammatory and are enriched in healthy people compared to patients with IBD,^55,56^ but we have not seen their association with anti-TNF. A recent study came to a similar conclusion that SCFAs are not the main pathways associated with remission, in this case after intervention with exclusive enteral nutrition.^58^

### 4.4. Strengths of the study

To the best of our knowledge, our study design may be unique in the parallel observation of patients with JIA on anti-TNF therapy—this enabled us to filter out the potential direct general effect of anti-TNF on any gut microbiome, including that of gut-healthy individuals. Moreover, the absence of mucosal inflammation in JIA was verified by faecal calprotectin testing, although this was a priori very unlikely in the absence of gastrointestinal symptoms.^64,65^ In accordance with recent trends, we used longitudinal observation to decrease the effects of short-term fluctuations in the abundance of individual microbial taxa.^66^ Our multi-centre patient recruitment might increase the generalizability of our results. Furthermore, we meticulously checked the sample integrity throughout the cold chain: the temperature in the study subject’s home freezers was checked, and shipping containers were frozen phase change cold packs [with holes for collection tubes] in a styropore box. Lastly, we obtained parallel calprotectin levels from all faecal samples, enabling us to adjust models for mucosal inflammation activity.

### 4.5. Limitations and their remedies

The foremost limitation is the limited number of JIA patients recruited into the study. In addition, the diagnosis itself is infrequent, and the children lacked the willingness to collect stool samples. The data, however, did not indicate any borderline effects of anti-TNF on either the JIA faecal metabolome or bacteriome: the difference in effect in contrast to CD is clear. To show that JIA patients do not differ in the overall faecal composition to control subjects, we used an older control dataset [age limit of 21 years, and we included first-year medical students in addition to paediatric controls]; as the goal was to document the absence of departure of JIA from the main cluster of control bacteriomes, this age difference is not likely to be important [Figure 2]. Additionally, entry to the study was not identical as two entry points were possible [Supplementary Figure 1].

### 4.6. Conclusions

We have described a shift in the faecal bacteriome and metabolome towards healthier compositions after anti-TNF therapy in CD, supported by a comprehensive longitudinal collection of stool samples. Most importantly, in a model adjusted for faecal calprotectin levels, we identified four bacterial taxa [notably SCFA producers *Flavonifractor* and *Ruminococcus*] and four faecal metabolites [especially isoleucine], whose abundance changed significantly after anti-TNF therapy only in CD and not in gut-healthy JIA. We observed no appreciable *in vitro* effect of infliximab on the growth of representative anaerobic bacteria, which, together with the above, implies that changes in the faecal bacteriome and metabolome are of secondary origin and that they probably reflect a decrease in mucosal inflammation rather than a general effect of anti-TNF. Consequently, the identified bacterial taxa and faecal metabolites could be seen as signs of a restored gut ecosystem, which might help to understand the nature of gut microbiome changes in during CD therapy.

## Supplementary Material

jjad126_suppl_Supplementary_Materials

jjad126_suppl_Supplementary_Table_Metabolites

## Data Availability

The data are available from authors upon reasonable request. Sequencing data along with essential metadata have been deposited in the Sequence Read Archive [SRA] at the National Center for Biotechnology Information [NCBI] database under accession number PRJNA958468.
