# Supplementary material for: Faecal Bacteriome and Metabolome Profiles Associated with Decreased Mucosal Inflammatory Activity Upon Anti-TNF Therapy in Paediatric Crohn’s Disease
Source: J Crohns Colitis. 2023 Aug 1;18(1):106–20. doi: 10.1093/ecco-jcc/jjad126 (PMC10821711; doi:10.1093/ecco-jcc/jjad126)
Supplement: jjad126_suppl_Supplementary_Materials [file jjad126_suppl_supplementary_materials.docx]

**Supplementary Methods**

**1. Stool samples collection and faecal calprotectin analysis**

The self-administered collection kits were distributed along with detailed instructions. First, subjects were instructed to defecate onto clean collection paper (Faeces Collection Paper Fe-Col, Alpha laboratories, UK) installed over the toilet seat. A walnut-sized piece of stool was then collected into a clean plastic tube using a spoon attached to the lid. The tube was marked using an enclosed pen marker, put in a pre-frozen transport container (Sarstedt, Germany) and stored in a home freezer. The subject's parents were instructed to use a regular thermometer (provided in the collection kit) and regulate the freezer temperature to at least -18°C. After three such collections, samples were transported in the container to the clinic within a regular visit.

Faecal calprotectin was measured by using a quantitative enzyme-linked immunosorbent assay EliA™ on the Phadia 250 machine (both Phadia AB, Sweden) according to the manufacturer's instructions. All measurements were done in an accredited laboratory (ISO 9001:2015).

**2. Analysis of the bacterial 16S rDNA profiles**

*2.1. Amplicon sequencing*

Bacteriome profiling was performed by sequencing the V4 region of the bacterial 16S rRNA gene according to Kozich et al. ^1^ The amplification primers were provisioned with adapters and indexes for the identification of samples; thus, the procedure involved only one round of PCR. Each DNA sample was amplified in duplicate. The samples were sequenced along with several replicates of DNA from the Microbial Mock Community (HM-276D, BEI Resources, USA) and with negative controls (PCR grade water instead of DNA). The PCR reaction contained 18 µl AccuPrime™ *Pfx* SuperMix (Invitrogen, USA), 1,2 µl of each primer (10 µM) and 1,2 µl of extracted DNA. The cycling conditions were: initial denaturation at 95 °C for 5 min, 30 cycles of 95 °C for 15 s, 55 °C for 30 s and 68 °C for 1 min, followed by a final step at 68 °C for 5 min. The amplified indexed DNA fragments were purified using Agencourt® SPRIselect (Beckman Coulter, USA), and their concentrations were measured using Qubit™ dsDNA HS Assay Kit (Invitrogen, USA) and pooled in identical molar quantities, or the purification and equalisation were carried out in a single step using the SequalPrep normalisation plates (Thermo, USA). The final pool of 2000 pM concentration was sequenced on a Miseq instrument using the version 2 2x250 cycle kit (Illumina, USA).

*2. 2. Bioinformatic analysis*

The ensuing demultiplexed *fastq* files with sequencing reads were downloaded and processed using the DADA2 pipeline (version 1.22) as advised by software authors.^2^ Sequences were taxonomically classified using SILVA database version 138.^3^ Amplicon sequence variants (ASVs, analogous to operational taxonomic units, OTUs) were then further analysed using vegan ^4^ and phyloseq ^5^ in the R programming language ^6^.

Samples were analysed in duplicates. First, we removed several failed reactions (with less than 1000 reads per replicate), and to verify the agreement between replicates, we inspected an ordination plot created by nonmetric multidimensional scaling of the Bray-Curtis dissimilarity. Then the duplicates were merged. Negative controls were checked for the absence of a significant signal, and the mock community reactions were assessed for agreement with their declared content. The read sets were then rarefied to 10 thousand read pairs per sample (close to the lowest read count per sample).

**3. Stool metabolome profiling**

*3.1. Processing stool sample for nuclear magnetic resonance (NMR) analysis*

Stool aliquot (200 mg) was transferred to a microtube. Then, 800 μl of ultrapure water was added and was then vortexed (3,000 rpm, 10 sec) and centrifuged (17,000 ×*g*, 10 min) using a fixed angle rotor. The resulting supernatant (540 μl) was transferred to another 1.5 ml microtube, and 60 μl of phosphate-buffer (1.5M K_2_HPO_4_ /1.5M NaH_2_PO_4_, 5 mM 3-(Trimethylsilyl) propionic-2,2,3,3-*d*_4_ acid sodium salt (TSP) + D_2_O, 0.2% NaN_3_, pH 7.4) solution was added. All chemicals were from Merck (NJ, USA). The sample was then centrifuged (17,000 ×*g*, 10 min) using a fixed angle rotor.

*3.2. Spectra processing*

Spectra were reduced to predefined spectral bins included in the region between δ_H_ 0.05 to δ_H_ 9 ppm, considering peak shape for segment definition with bin borders set in baseline regions and calculated using approximation of the integration over an interval by breaking the area down into trapezoids. Each bin represented a spin system or a part of a spin system that was ideally pure, distinct, and quantitative for the given metabolite. One bin represented one metabolite but, in some cases, for metabolites overlapping with other signals, multiple bins were chosen for reference. Ranges for the bins were chosen after previous annotation of a subset of spectra in software Chenomx ver. 8.6, using build-in spectral library and our in-house database. The regions corresponding to water (δ_H_ 4.70–5.15 ppm) were excluded from the analysis. Thus, the bins represented integrals of 57 unique annotated compounds, which were corrected for dilution factor using probabilistic quotient normalisation. Raw data and normalized data together with bin ranges in δ (ppm) are listed in **Supplementary table - Metabolites**.

**Supplementary Table 1.** Paris classification of Crohn's disease ^i^

| **Status** | **Total** | Recieved anti-TNF | Without anti-TNF |
| --- | --- | --- | --- |
| N of subjects | **54** | 37 | 17 |
| **Localisation** |  |  |  |
| L1: Terminal ileal+/- limited caecal, n (%) | **7**  **(13%)** | 5  (14%) | 2  (12%) |
| L2: Colonic,  n (%) | **10**  **(19%)** | 5  (14%) | 5  (29%) |
| L3: Ileocolic,  n (%) | **37**  **(69%)** | 27  (73%) | 10  (59%) |
| L4a or L4b*,  n (%) | **30**  **(56%)** | 21  (57%) | 9  (53%) |
| **Behaviour** |  |  |  |
| B1: non-stricturing non-penetrating,  n (%) | **45**  **(83%)** | 32  (86%) | 13  (76%) |
| B2: stricturing, n (%) | **9**  **(17%)** | 5  (14%) | 4  (24%) |
| B3:penetrating,  n (%) | **0** | 0 | 0 |
| **Perianal disease, n (%)** | **11**  **(20%)** | 11  (30%) | 0 |
| **Growth impairment, n (%)** | **5**  **(9%)** | 3  (8%) | 2 (12%) |

**^i^** In Paris Classification systems L4 and L4a/L4b may coexist with L1, L2, and L3, respectively.

**Supplementary Table** **2.** Bacterial taxa with significant abundance shift in CD patients upon anti-TNF administration: graphic representation.

| **Bacterial taxon** | **Percent point change in CD patients upon administering anti-TNF** | **Abundance relative to the date of first anti-TNF dose (or to the last available sample in subjects not receiving the therapy)** | **Bacterial abundance from the multilevel model, stratified by the clinical response assessed by MINI index** |
| --- | --- | --- | --- |
| p. Bacteroidota  └─ c. Bacteroidia  └─ o. Bacteroidales | -8.1 |  | 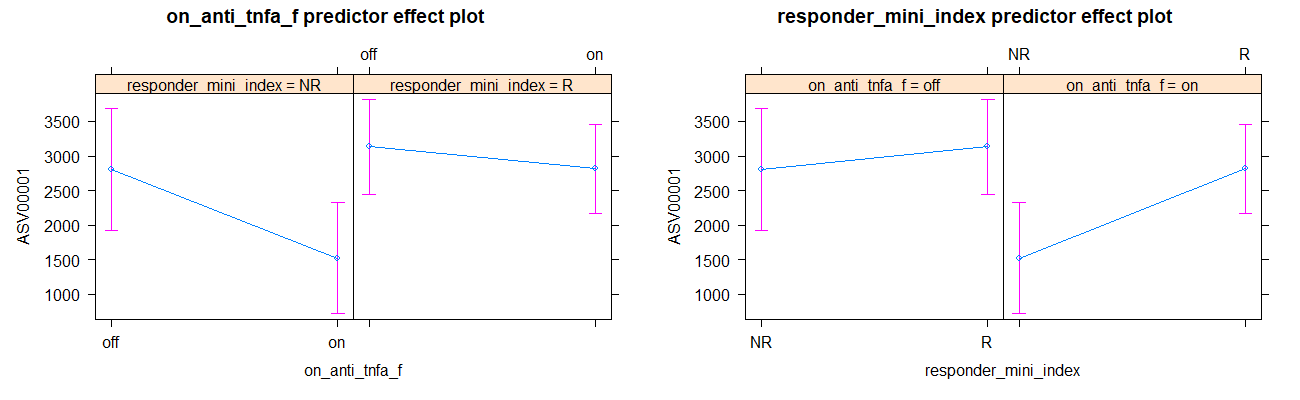 |
| └─ f. Rikenellaceae  │ | -1.9 |  | 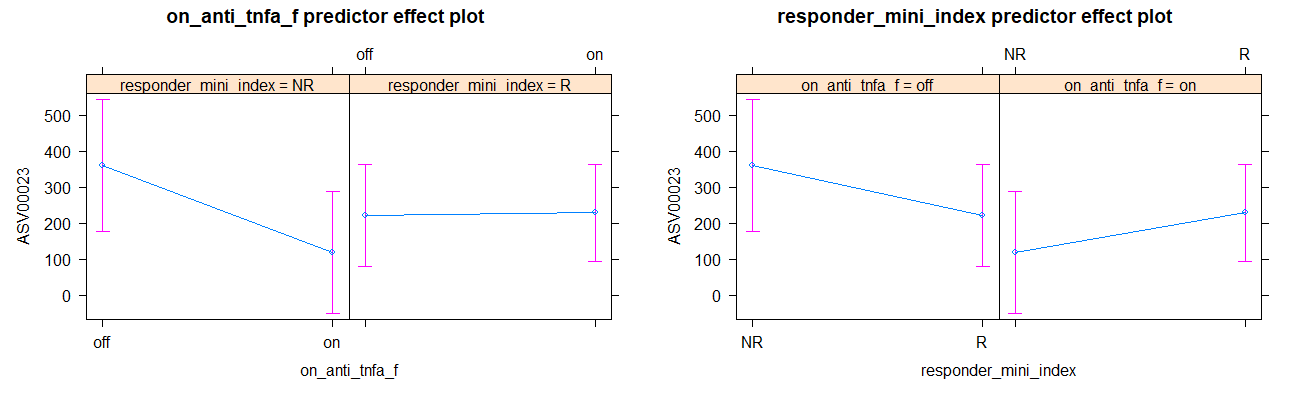 |
| └─ g. *Alistipes* | -2.0 |  | 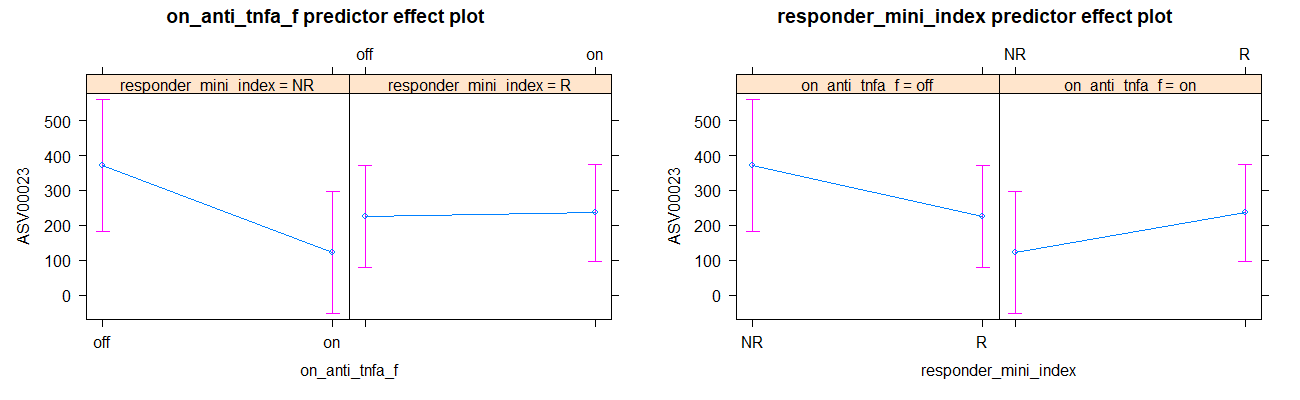 |
| p. Firmicutes | +9.7 |  | 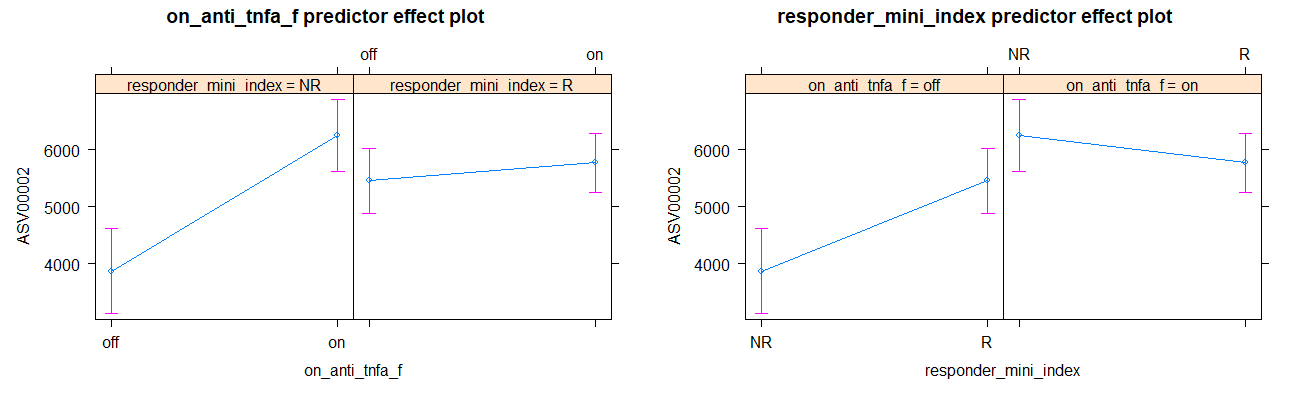 |
| └─ c. Clostridia  │ | +11 |  | 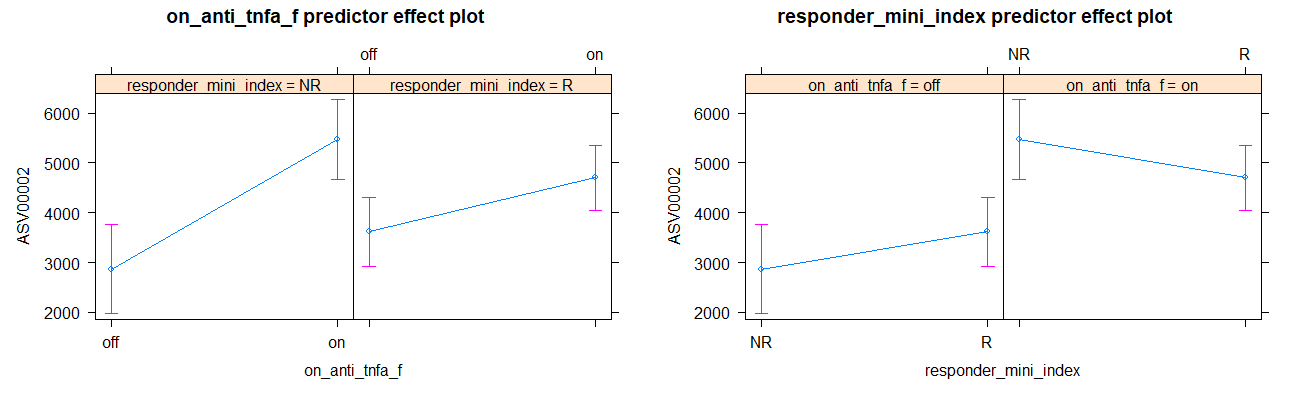 |
| │─ o. Peptostreptococcales  │ | +1.4 |  | 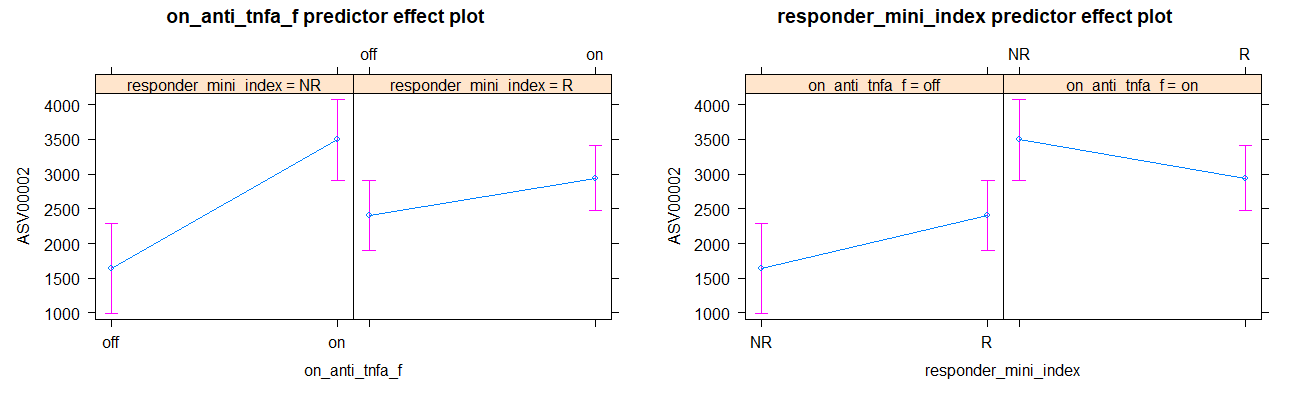 |
| │└─ f. Peptostreptococcaceae  │ | +1.1 |  | 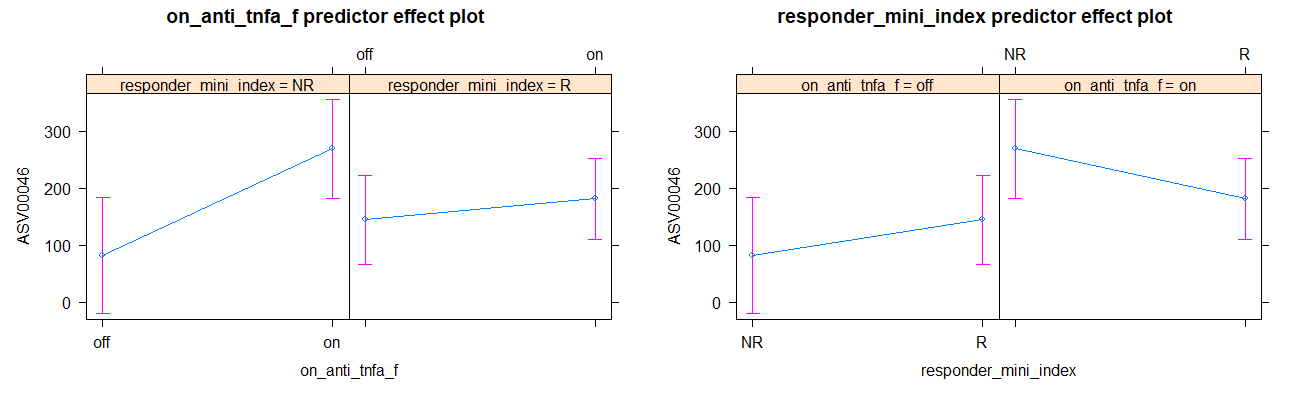 |
| │ └─ g. *Intestinibacter*  │ | +0.75 |  | 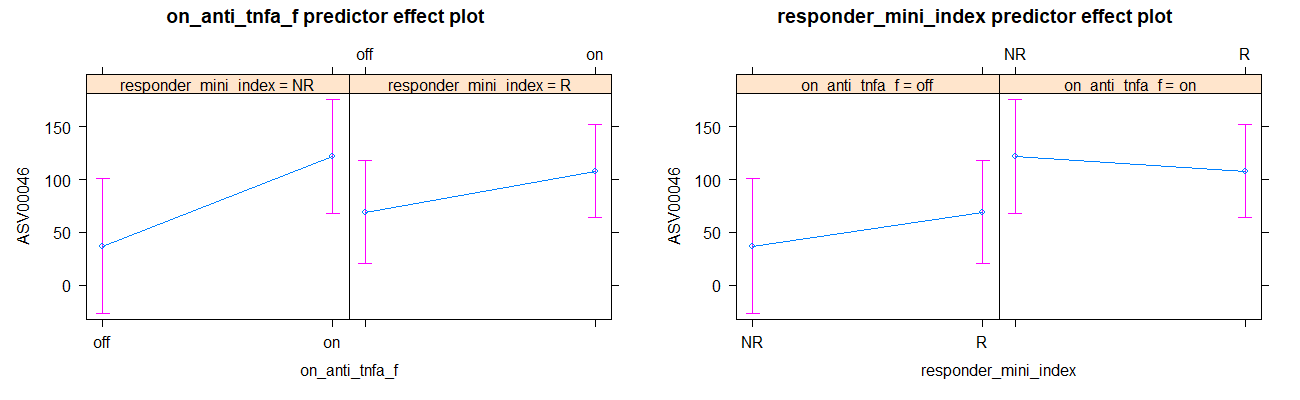 |
| │─ o. Lachnospirales  │ | +9.4 |  | 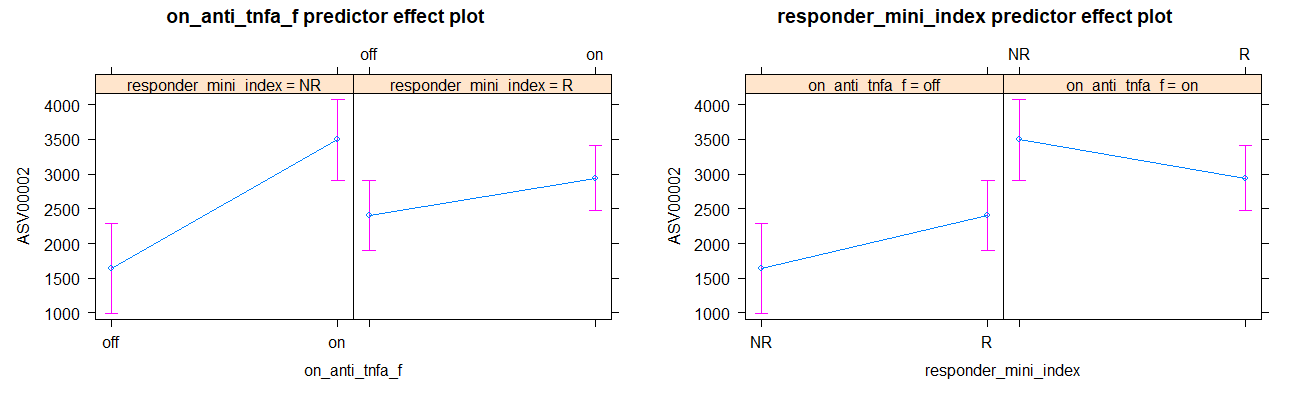 |
| │ └─ f. Lachnospiraceae  │ | +9.4 |  | 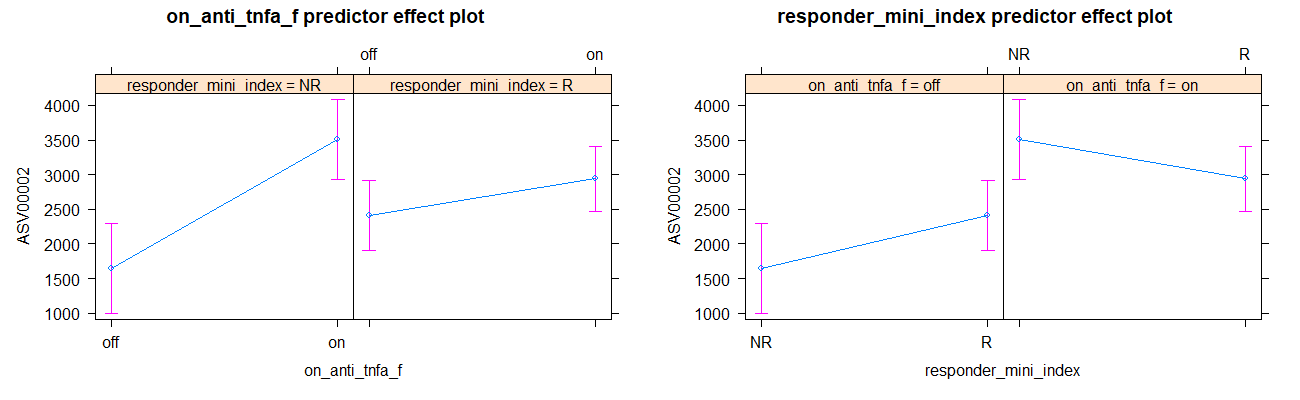 |
| │ └─ g. *Ruminococcus*  │ | +2.7 |  | 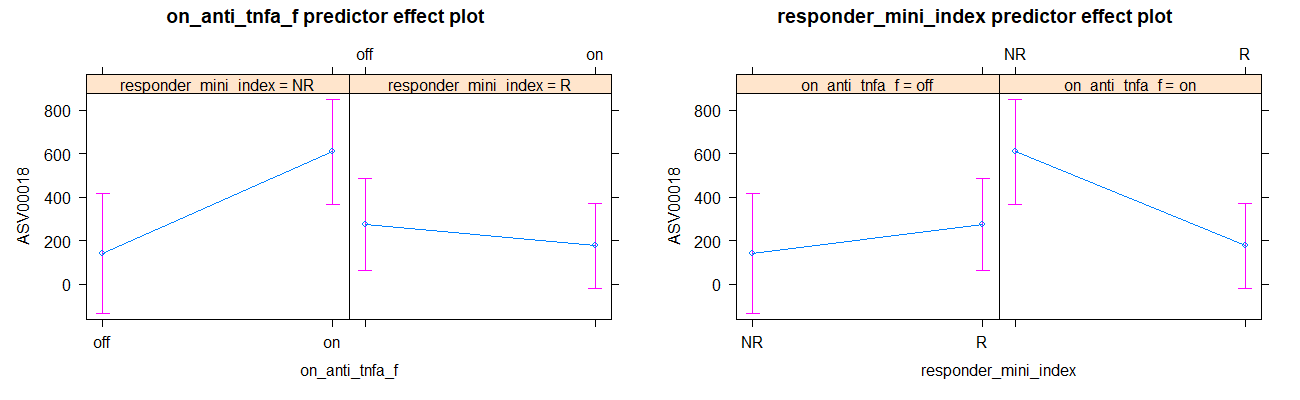 |
| └─ ..... g. *Flavonifractor* | +0.38 |  | 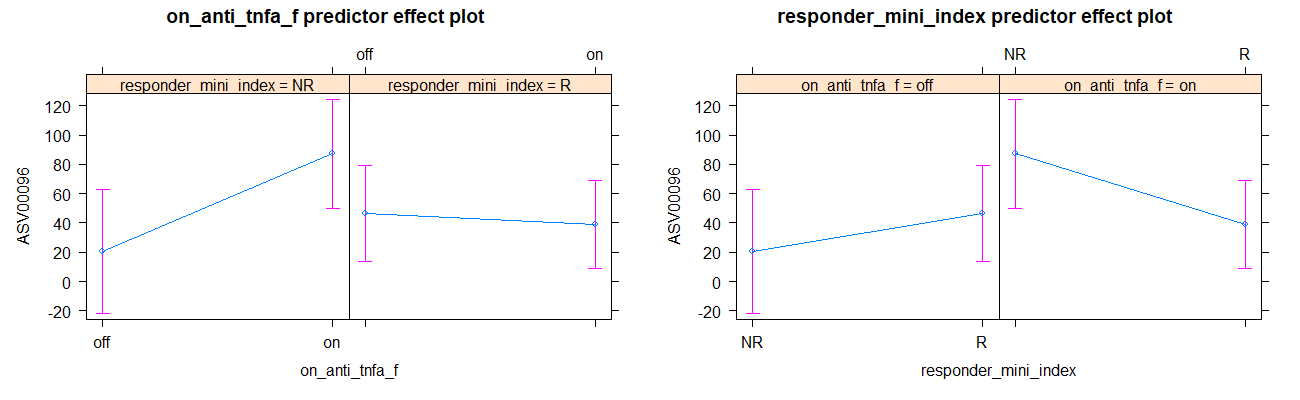 |

Left panel:

Trend curves with 95% confidence intervals are plotted using all samples, so their slopes, intercepts and standard deviations do not reflect the repeated sampling.

Right panel:

Estimated mean quantities of the taxon are shown from multilevel models with random intercept for subject. They are plotted by responder status in MINI index at 3 months after the commencement of anti-TNF-alpha therapy, before (off) and on the therapy.

CD, Crohn’s disease; IBD, here in figures Crohn disease; JIA juvenile idiopathic arthritis

**Supplementary Table 3**. Principal components of the bacteriome at the phylum level, and their associations

| **Principal component** | **PC1** | **PC2** | **PC3** |
| --- | --- | --- | --- |
| Explained percentage of variance | 47% | 24% | 13% |
| Cumulative percentage of variance | 47% | 71% | 84% |
| Loadings of main phyla |  |  |  |
| Association in JIA with anti-TNF therapy | NS | NS | NS |

| Crohn's disease: associations   - with time - with calprotectin - with anti-TNF therapy | NS  NS  decrease upon therapy (P=1.9x10^-4^) | direct association (P=3.7x10^-4^)  inverse association (P=1.7x10^-5^)  NS | NS  inverse association (P=8.6x10^-5^)  NS |
| --- | --- | --- | --- |
| Crohn's disease, change in the PC  after anti-TNF therapy   - the component baseline differs responder vs nonresponder - the change in component with anti-TNF therapy - intensity of change in PC differs by responder/nonresponder | NS  A decrease in PC1 upon therapy (P=3.0x10^-5^)  Less pronounced decrease observed in responders (P=6.9x10^-3^) | Baseline higher in responders (P=0.017)  An increase in PC2 upon therapy  (P=5.2x10^-4^)  NS | NS  NS  NS |

**Supplementary Figure 1.** Study scheme


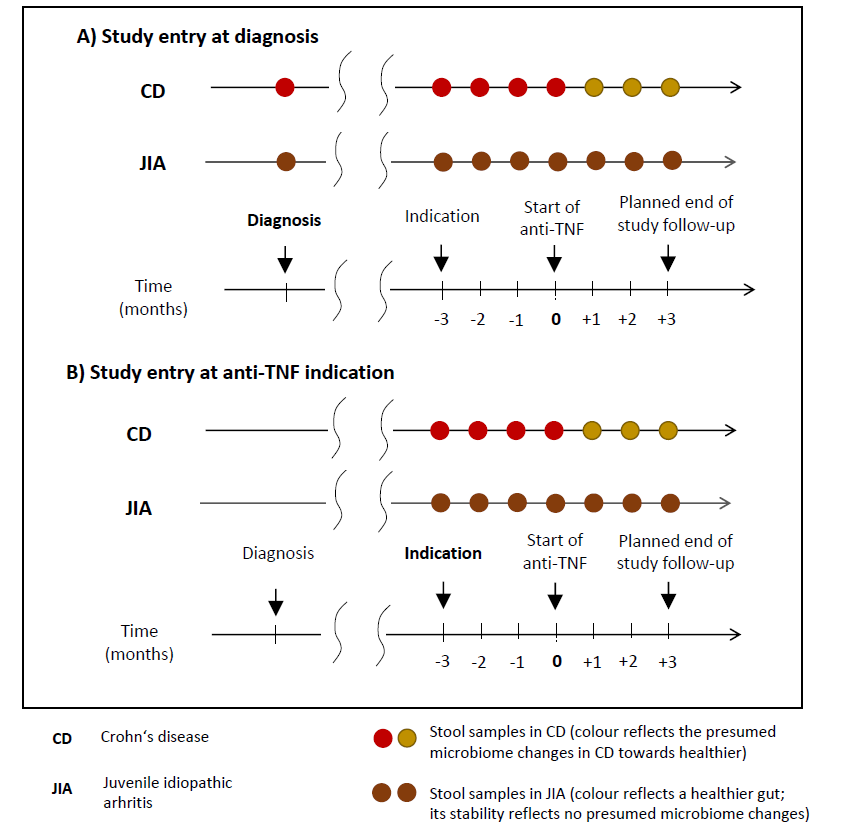


**Supplementary Figure 2.** Stool samples in time


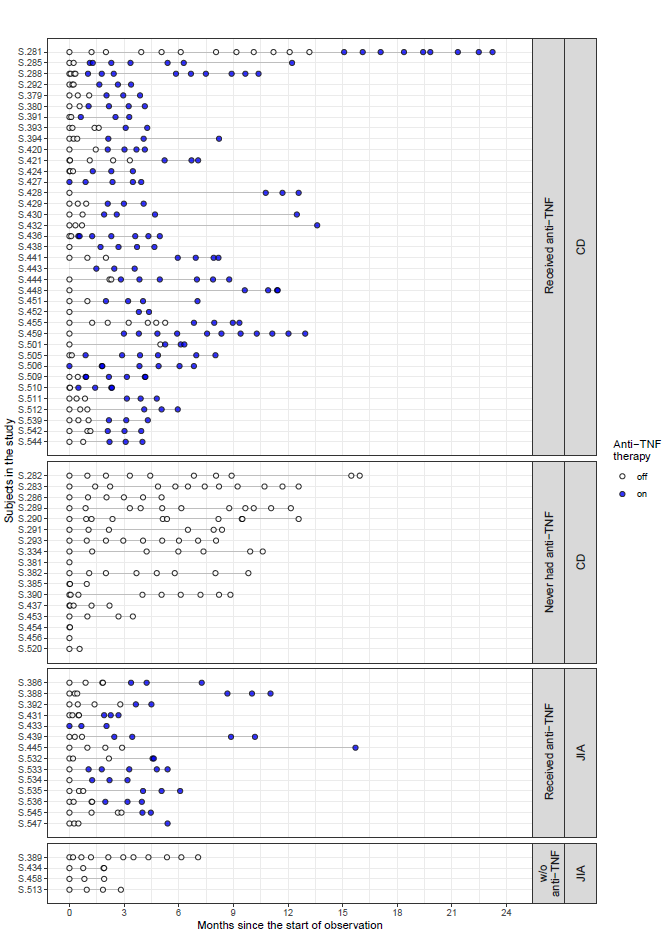
blue = samples on therapy, white = samples taken while patient is off anti-TNF therapy

**Supplementary Figure 3.** The time trends of calprotectin levels by diagnosis and anti-TNF therapy


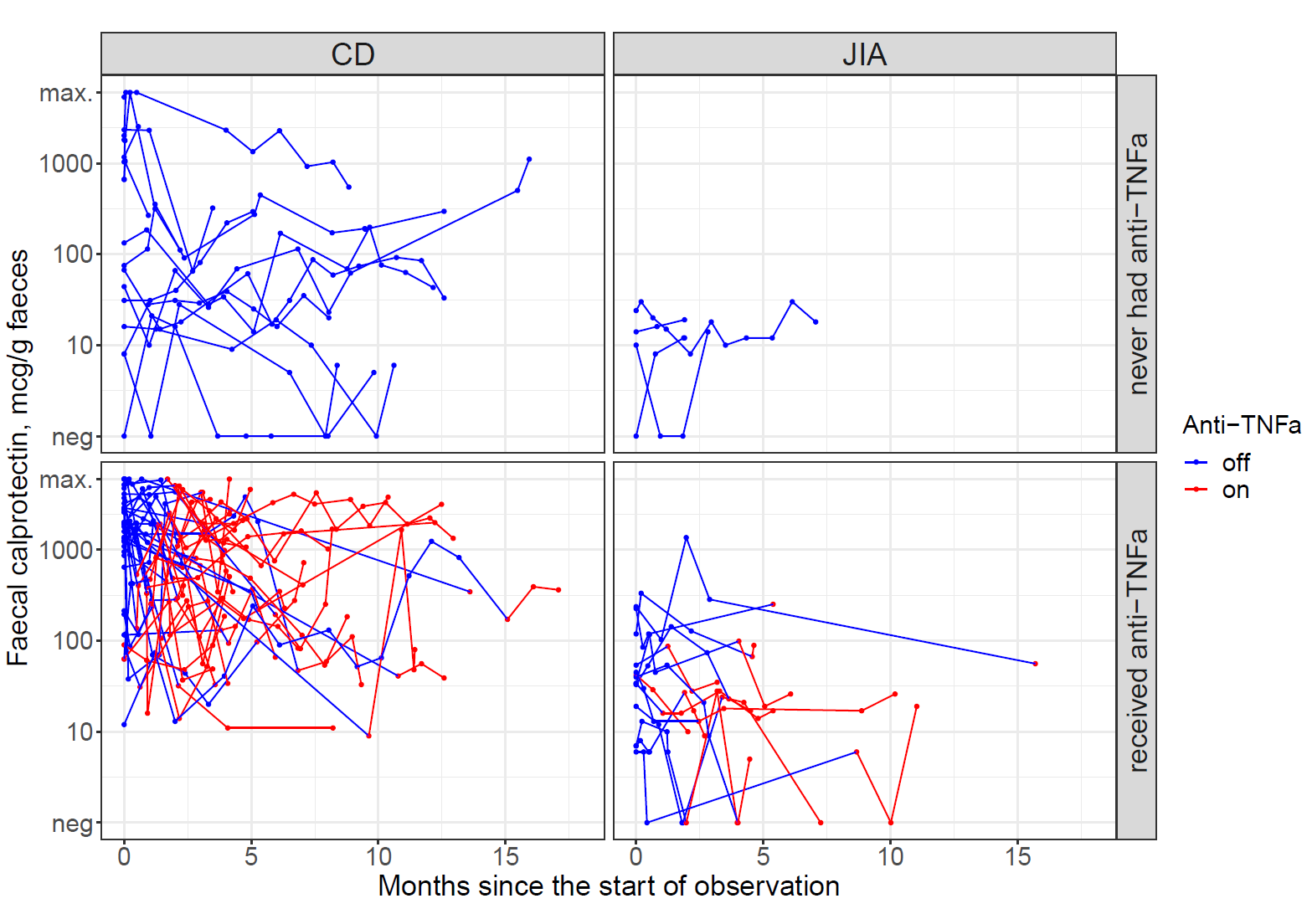


Calprotectin levels are plotted on a logarithmic scale. Currently, there is no ideal cut-off point for monitoring faecal calprotectin. Usually, values under 50-100 are considered normal, and values over 250 mcg/g are pointing to gut inflammation. ^7, 8^

The single outlier in the JIA group who received the treatment is a sample of a subject who had a COVID-19 infection at the time of sampling. All the other samples of this subject were within the normal range.

Blue = samples off anti-TNF therapy; red = samples on anti-TNF therapy

**Supplementary Figure 4.** Bacteriome alpha diversity indices by diagnosis

CTR = healthy control, JIA = Juvenile idiopathic arthritis, CD = Crohn’s disease

**Supplementary Figure 5.** Alpha diversity changes with time in CD but not in JIA.


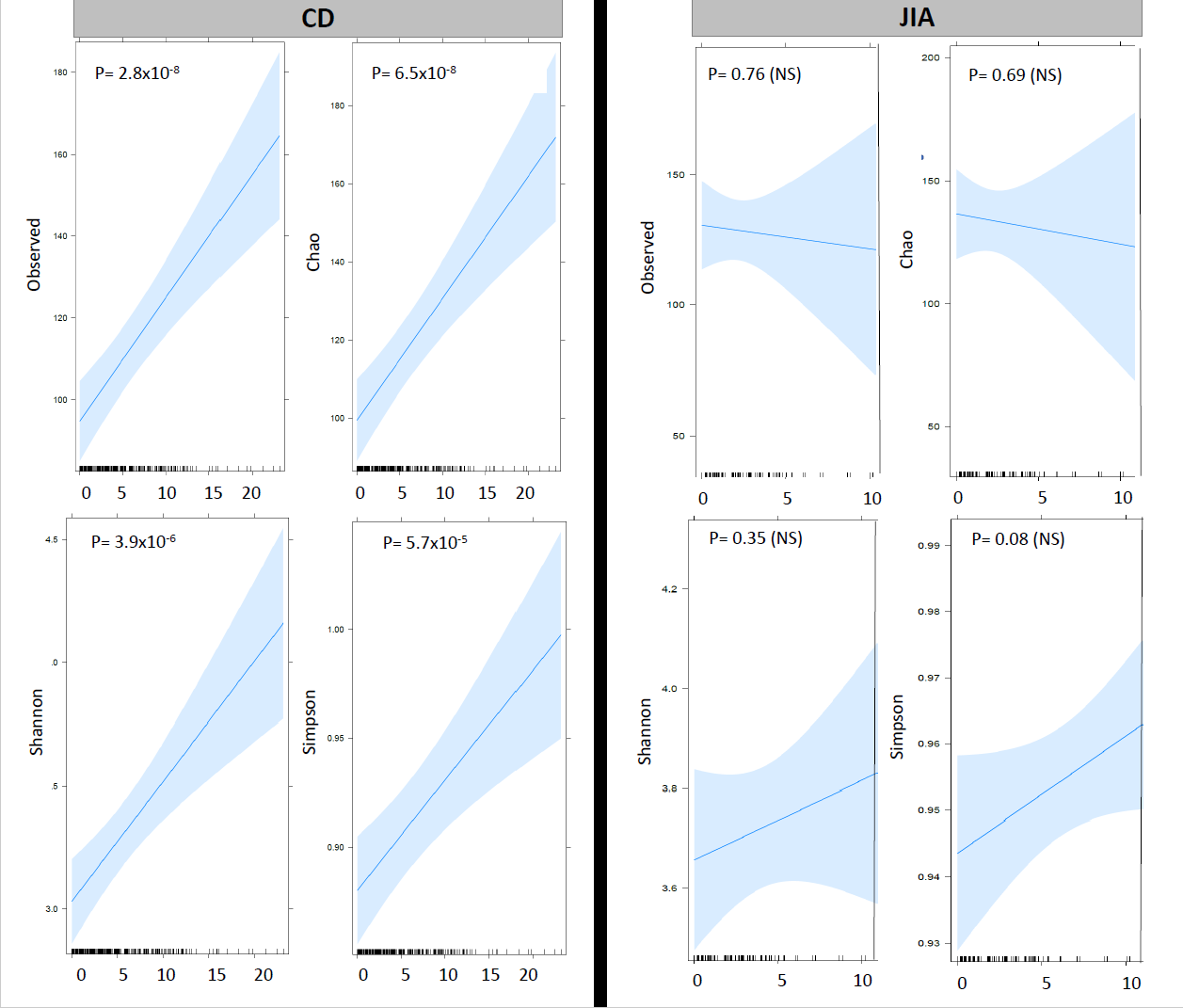


Coefficients and prediction bands from a generalized estimating equations model are used to show the changes of four alpha diversity indices (vertical axis) with time in months (horizontal axis) in CD (left two panels) and in JIA (right two panels).

NS = non-significant; CD = Crohn’s disease; JIA = Juvenile idiopathic arthritis

**Supplementary Figure 6.** PCA ordination plots and the abundance of important phyla in the first three PCA axes

**(A) PCA axes 1 and 2 and their main phyla**

**(B) PCA axes 2 and 3 and their main phyla**

**(C) Alpha diversity decreases along with decreasing PC2 score, and to a lesser extent also with decreasing PC1 score.**

The association of alpha diversity indices was particularly pronounced for PCA axis 1 (highly significant for all indices, P<0.001), whereas PCA axis 2 scores were highly significant with Shannon and Simpson indices (P<10^-6^), but less pronounced for the remaining indices (P<0.03).

**(D) Calprotectin levels plotted against the first three PCA axes**

**Supplementary Figure 7.** Metabolome PCA, first two axes.

**Supplementary Figure 8.** Metabolites associated with anti-TNF therapy.

Relative abundance of faecal metabolites associated with the anti-TNF therapy in CD. Metabolites with corrected P value for the anti-TNF term are shown by diagnosis and therapeutic group. Units on the vertical scale are normalized areas under the spectra, and are not comparable among metabolites.


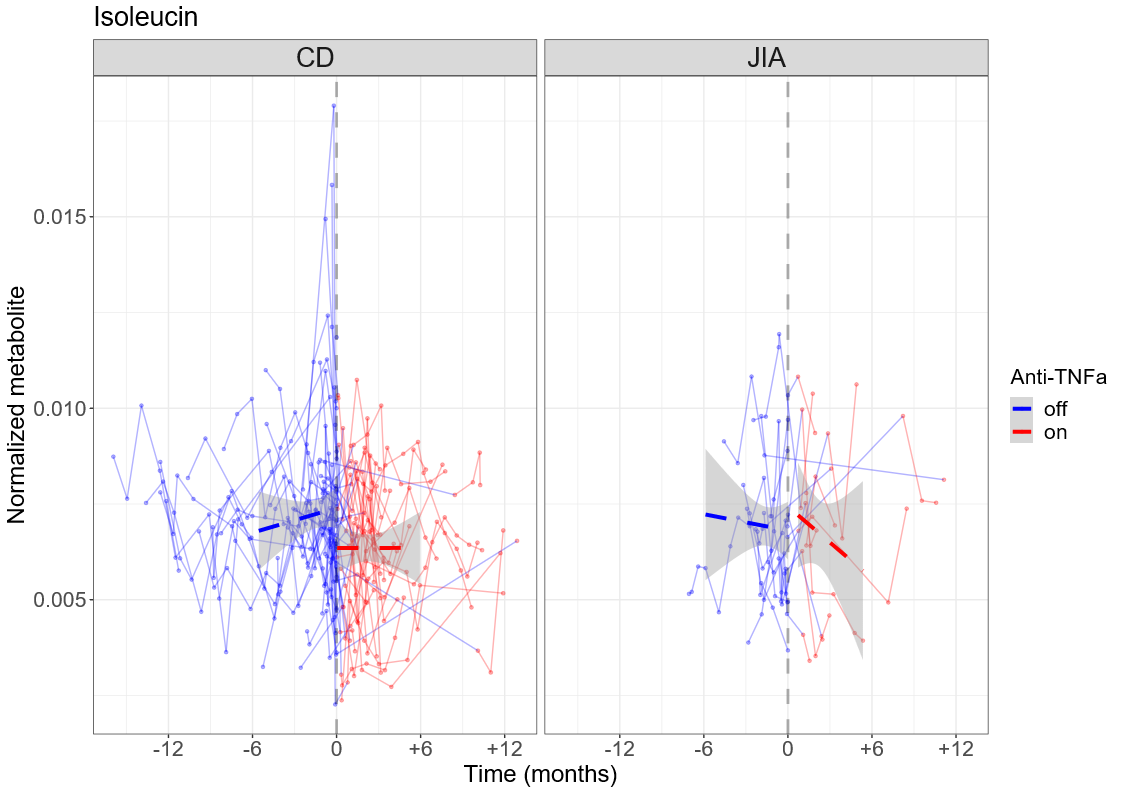


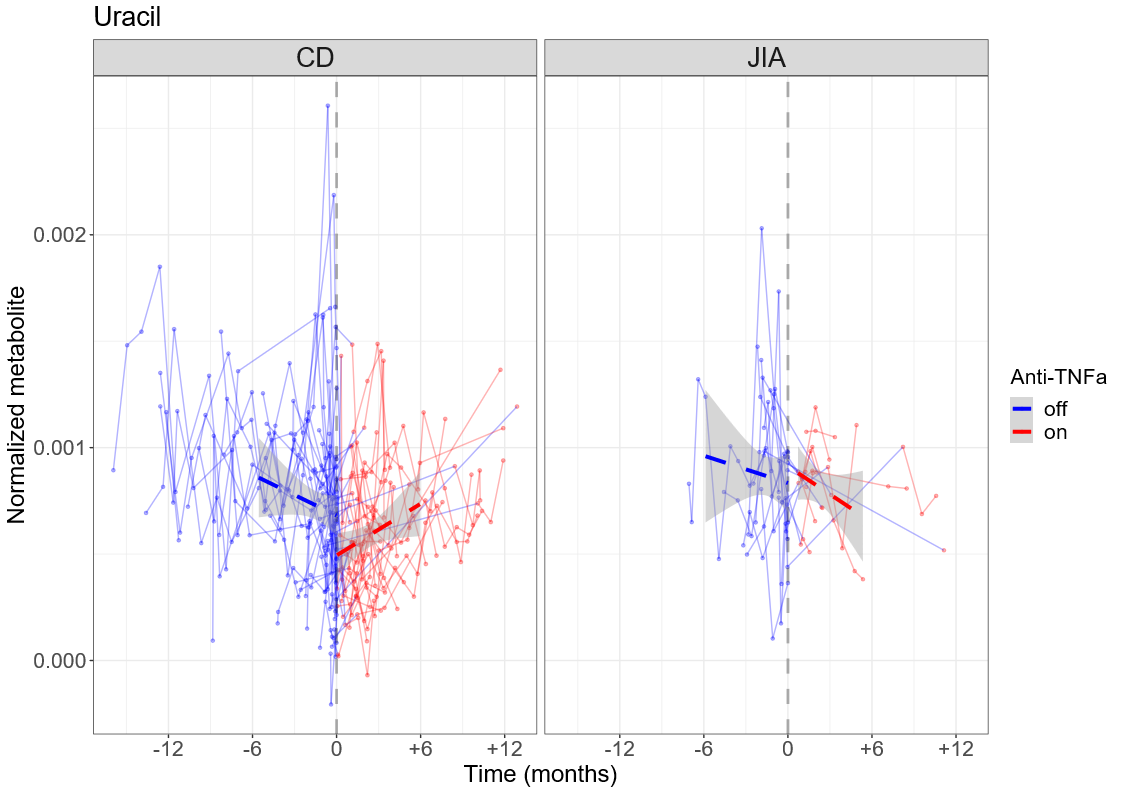


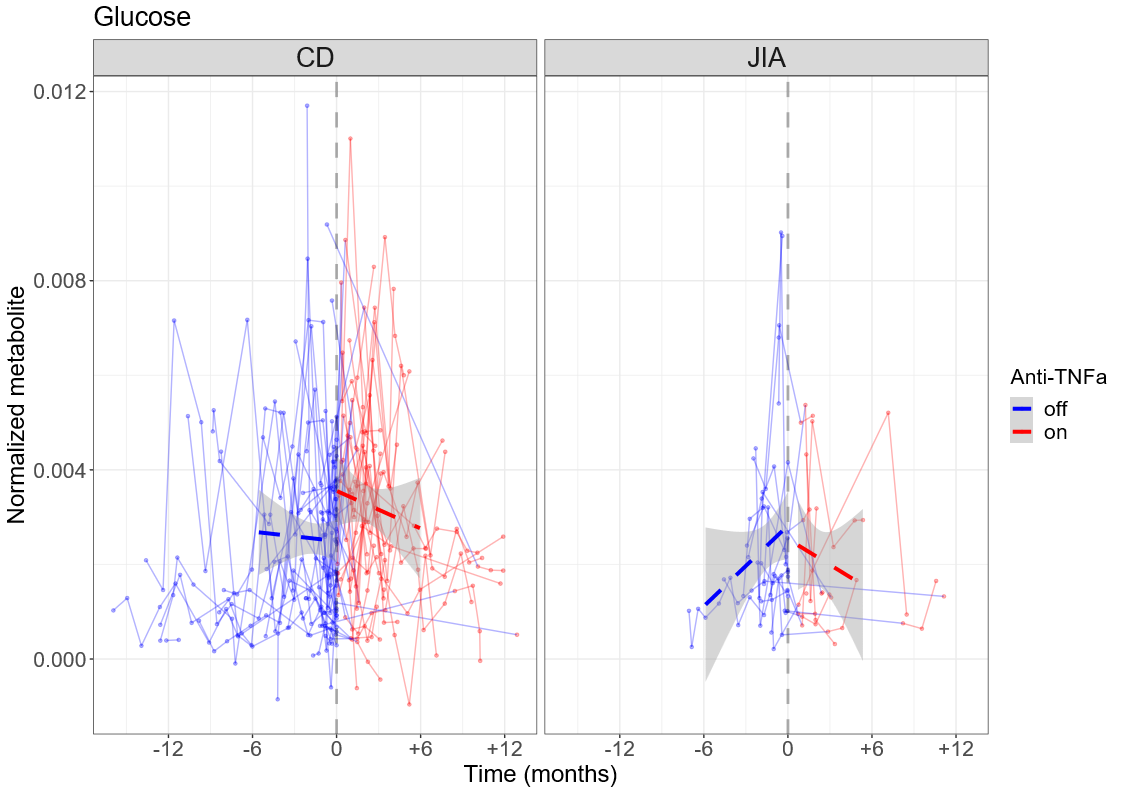


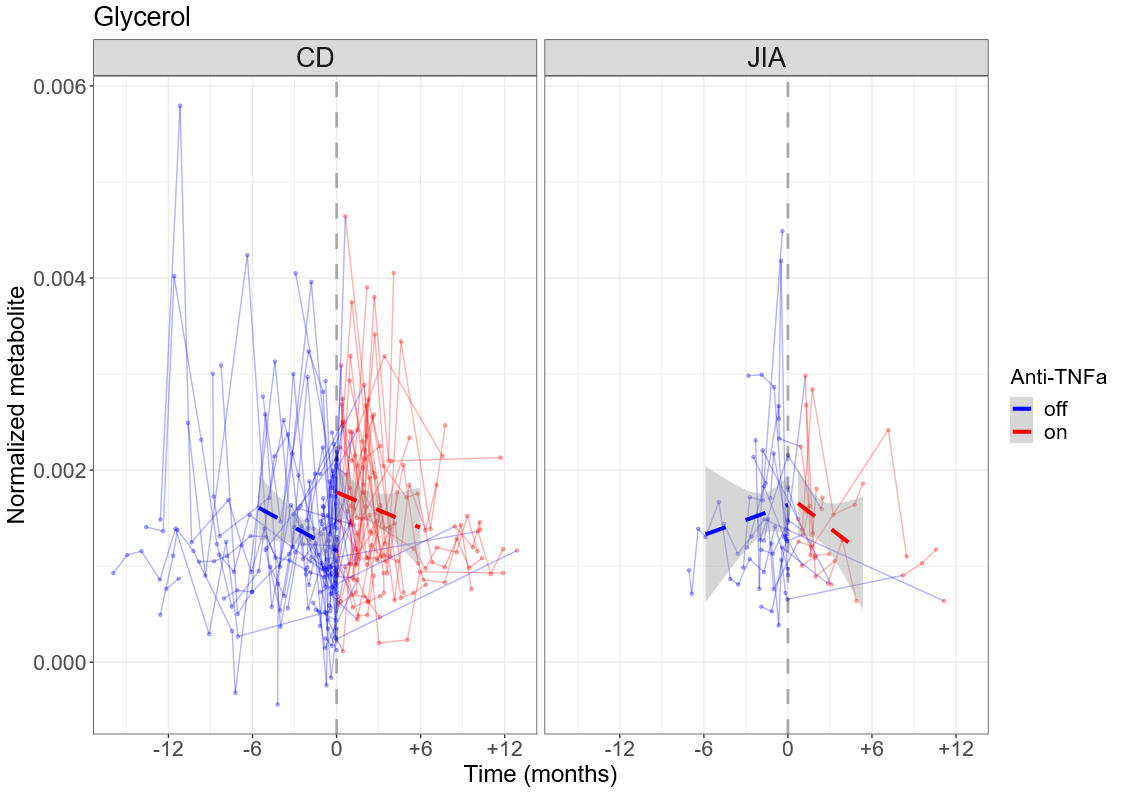


**Supplementary Figure 9.** Co-inertia analysis of stool bacteriome and stool metabolome profiles.

Metabolome profiles (left) are compared to bacteriome profiles at the level of genus. The two tables are significantly correlated (P < 0.0001)


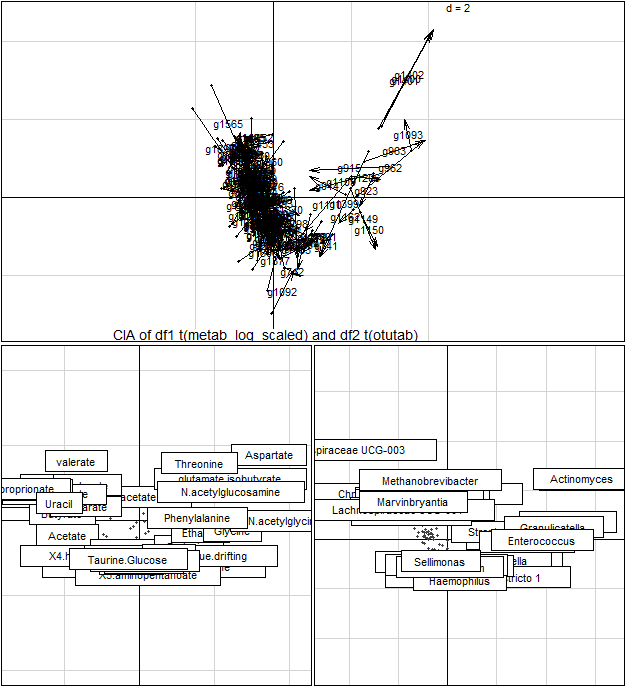


**Supplementary References**

1. Kozich JJ, Westcott SL, Baxter NT, et al. Development of a dual-index sequencing strategy and curation pipeline for analyzing amplicon sequence data on the MiSeq Illumina sequencing platform. Appl Environ Microbiol 2013;79:5112-20.

2. Callahan BJ, McMurdie PJ, Rosen MJ, et al. DADA2: High-resolution sample inference from Illumina amplicon data. Nat Methods 2016;13:581-3.

3. Quast C, Pruesse E, Yilmaz P, et al. The SILVA ribosomal RNA gene database project: improved data processing and web-based tools. Nucleic Acids Res 2013;41:D590-6.

4. Oksanen J, Guillaume Blanchet F, Friendly M, et al. vegan: Community Ecology Package. R package version 2.5-6., 2019.

5. McMurdie PJ, Holmes S. phyloseq: an R package for reproducible interactive analysis and graphics of microbiome census data. PLoS One 2013;8:e61217.

6. Team RC. R: A language and environment for statistical computing. 3.4.2 ed. Vienna, Austria: R Foundation for Statistical Computing, 2017.

7. Maaser C, Sturm A, Vavricka SR, et al. ECCO-ESGAR Guideline for Diagnostic Assessment in IBD Part 1: Initial diagnosis, monitoring of known IBD, detection of complications. J Crohns Colitis 2019;13:144-164.

8. D'Amico F, Nancey S, Danese S, et al. A Practical Guide for Faecal Calprotectin Measurement: Myths and Realities. J Crohns Colitis 2021;15:152-161.
